# Parallel clinal variation in the mid-day siesta of Drosophila melanogaster implicates continent-specific targets of natural selection

**DOI:** 10.1371/journal.pgen.1007612

**Published:** 2018-09-04

**Authors:** Yong Yang, Isaac Edery

**Affiliations:** 1 Rutgers University, Center for Advanced Biotechnology and Medicine, New Jersey, United States of America; 2 Department of Molecular Biology and Biochemistry, Rutgers University, Center for Advanced Biotechnology and Medicine, New Jersey, United States of America; Washington University in Saint Louis School of Medicine, UNITED STATES

## Abstract

Similar to many diurnal animals, *Drosophila melanogaster* exhibits a mid-day siesta that is more robust as ambient temperature rises, an adaptive response aimed at minimizing exposure to heat. Mid-day siesta levels are partly regulated by the thermosensitive splicing of a small intron (termed dmpi8) found in the 3’ untranslated region (UTR) of the circadian clock gene *period* (*per*). Using the well-studied *D*. *melanogaster* latitudinal cline along the eastern coast of Australia, we show that flies from temperate populations sleep less during the day compared to those from tropical regions. We identified combinations of four single nucleotide polymorphisms (SNPs) in the 3’ UTR of *per* that yield several different haplotypes. The two most abundant of these haplotypes exhibit a reciprocal tropical-temperate distribution in relative frequency. Intriguingly, transgenic flies with the major tropical isoform manifest increased daytime sleep and reduced dmpi8 splicing compared to those carrying the temperate variant. Our results strongly suggest that for a major portion of *D*. *melanogaster* in Australia, thermal adaptation of daily sleep behavior included spatially varying selection on ancestrally derived polymorphisms in the *per* 3’ UTR that differentially control dmpi8 splicing efficiency. Prior work showed that African flies from high altitudes manifest reduced mid-day siesta levels, indicative of parallel latitudinal and altitudinal adaptation across continents. However, geographical variation in *per* 3’ UTR haplotypes was not observed for African flies, providing a compelling case for inter-continental variation in factors targeted by natural selection in attaining a parallel adaptation. We propose that the ability to calibrate mid-day siesta levels to better match local temperature ranges is a key adaptation contributing to the successful colonization of *D*. *melanogaster* beyond its ancestral range in the lowlands of Sub-Saharan Africa.

## Author summary

In warm climates many animals, including humans, exhibit a mid-day siesta, almost certainly a behavior meant to minimize the harm from prolonged exposure to the hot mid-day sun. But what about animals that adapted to cooler more temperate climates, might they have a less pronounced siesta? Indeed, we show that in the common fruit fly, *Drosophila melanogaster*, those from temperate regions in Australia exhibit less mid-day siesta compared to their tropical counterparts. Prior work showed that mid-day sleep levels are partially regulated by a ‘clock’ gene called *period* (*per*), which controls the timing of wake-sleep cycles in addition to other daily rhythms. We identified several DNA differences in the *per* gene that show geographical variation and contribute to the daytime sleep differences in flies from tropical and temperate regions via a mechanism that involves how well a temperature-sensitive intron in *per* is removed. A similar reduction in mid-day sleep was previously observed in African flies that adapted to the cooler temperatures found at high altitudes. Together, our findings provide a rare example where latitude and altitude lead to a similar behavioral adaptation to temperature. Moreover, the results suggest inter-continental differences in the evolutionary solutions used to attain the same thermal adaptation to cooler climates.

## Introduction

Daily wake-sleep cycles in animals are partially governed by interacting networks of cell-based circadian (≅24 hr) ‘clocks’ or pacemakers located in the brain, in addition to sleep homeostatic and arousal pathways. *Drosophila melanogaster* is an excellent animal model system to study the mechanisms underlying circadian rhythms and sleep [1–4]. Similar to many diurnal animals, the daily distribution of activity in *Drosophila melanogaster* exhibits a bimodal pattern with clock-controlled morning and evening bouts of activity separated by a dip in activity during the middle of the day, generally referred to as mid-day ‘siesta’ [5, 6]. Increases in average daily temperature are accompanied by a gradual delay in the onset of the evening bout of activity and a more prolonged mid-day siesta [7], presumably an adaptive response that minimizes the risks associated with exposure to the hot mid-day sun.

This thermal plasticity in daily behavior is partially controlled by the thermosensitive splicing of an intron in the 3’ untranslated region (UTR) of the *D*. *melanogaster period* (*d**per*) transcript [7, 8], which is the key circadian clock factor regulating the pace of the clock [9]. Removal of this short intron, named dmpi8 (*D*. *m**elanogaster* *p**er* intron 8), is progressively more efficient at cooler temperatures, which increases *dper* mRNA levels, somehow leading to reductions in mid-day siesta [7, 8, 10]. The thermosensitivity in dmpi8 splicing is based on suboptimal 5’ and 3’ splice sites (ss) [8], suggesting that at higher temperatures binding of the spliceosome is less efficient. Although it is not clear how splicing of dmpi8 and its associated effects on *dper* mRNA cycling regulate mid-day activity levels, recent findings indicate that it functions in a non-circadian role to modulate the daytime balance between sleep and wake [11]. For example, transgenic flies where the strengths of the 5’ and 3’ ss were increased, exhibit highly efficient dmpi8 splicing and reduced mid-day sleep compared to their wildtype controls [11]. This effect on mid-day sleep levels was mainly attributed to a decrease in sensory-mediated arousal thresholds with increased dmpi8 splicing efficiency [11]. While nighttime sleep levels are generally less affected by dmpi8 splicing compared to daytime sleep, it follows the same trend in being less consolidated as dmpi8 splicing efficiency increases [11, 12].

In contrast to other *Drosophila* species, *D*. *melanogaster* shows a wide spatial range beyond its ancestral origins in the lowlands of equatorial Africa, spreading to many continents and successfully colonizing cooler temperatures found in temperate climates and high altitudes [13–15]. We reasoned that if the thermal plasticity in mid-day sleep levels is an important attribute in the colonization of *D*. *melanogaster* to more temperate regions, it might show geographical variation. Specifically, adaptation to regions with cooler climates then those typical of its ancestral range might be accompanied by a reduction in baseline mid-day siesta levels as daily risks from heat exposure are reduced. Indeed, using natural populations of *D*. *melanogaster* from equatorial Africa, we recently showed that those adapted to high altitudes have less mid-day sleep levels over a broad range of temperatures compared to their lowland counterparts [12]. Moreover, for a large portion of flies from tropical Africa they showed altitudinal variation in dmpi8 splicing efficiency. The results suggest that selection for increased dmpi8 splicing efficiency played a major role in the thermal adaptation of African flies to the cooler climates found at high altitudes.

Intriguingly, there are several single nucleotide polymorphisms (SNPs) in the *dper* 3’ UTR that can modulate dmpi8 splicing efficiency and mid-day sleep without eliminating its thermal sensitivity [12, 16] (and see below). The presence of SNPs in the *dper* 3’ UTR that can affect dmpi8 splicing efficiency and mid-day siesta indicates that this splicing event contributes to natural variation in the sleep behavior of *D*. *melanogaster* in the wild. However, we did not observe any *dper* 3’ UTR SNP or SNP combination that might underlie altitudinal variation in dmpi8 splicing efficiency, nor that varied as a function of elevation [12]. A lack of latitudinal variation in the frequency of *dper* 3’ UTR SNPs or SNP combinations was previously noted for flies from the eastern coast of the United States [16], although for these populations of flies, latitude might preferentially affect nighttime sleep and not daytime sleep behavior [17]. Thus, despite the presence of multiple SNPs in the *dper* 3’ UTR, to date we have not found any evidence that they exhibit clinal variation or contribute to local adaptation.

Herein we extended our clinal studies of daytime sleep behavior by measuring daily wake-sleep patterns between opposite ends of a latitudinal cline of *D*. *melanogaster* from Australia that includes tropical (Queensland) and temperate (Tasmania) populations [18]. Prior studies showed that these two geographical extremes exhibit strong genomic and phenotypic variation that are thought to arise from spatially varying selection and not demographic history (e.g., [19–24]). Our results demonstrate that natural populations from temperate climates manifest lower levels of daytime sleep compared to those from tropical regions. The daily splicing efficiency of dmpi8 is higher in flies from temperate climates, consistent with the decreased daytime sleep. Thus, there is remarkable convergence in the thermal adaptation of daytime sleep as a function of altitude and latitude in two widely divergent populations representing ancestral populations in Africa and more recent cosmopolitan ones in Australia. However, for *D*. *melanogaster* from Australia, we identified two major *dper* 3’ UTR haplotypes that exhibit inverted directionality in their relative frequencies between tropical and temperate populations. Remarkably, the major tropical variant manifests lower dmpi8 splicing efficiency and increased mid-day siesta compared to the major temperate variant. Our results strongly suggest that for a large portion of *D*. *melanogaster* from Australia, thermal adaptation of mid-day sleep levels also includes spatially varying selection on ancestrally derived polymorphisms in the *dper* 3’ UTR that differentially calibrate dmpi8 splicing efficiency. We propose that the successful colonization of *D*. *melanogaster* beyond its ancestral range in the lowlands of Sub-Saharan Africa was facilitated on numerous occasions by its ability to calibrate mid-day siesta levels according to the relative risks associated with local temperature ranges, a parallel adaptation that has involved inter-continental differences in factors targeted by natural selection.

## Results

### Australian *D*. *melanogaster* from temperate regions exhibit decreased levels of daytime sleep and more fragmented overall sleep

Numerous studies have reported on genomic and phenotypic clinal variation in natural populations of *D*. *melanogaster* collected along the eastern coast of Australia (e.g., [19–23]). Unfortunately, many of the independent isofemale lines are no longer extant from the original sources (C. Sgro, personal communication). Nonetheless, we were able to acquire flies from a well-studied collection that represent the extreme ends of the cline, previously termed temperate (Tasmania; Sorell, 42.769^o^ S) and tropical (Queensland; Cairns, 16.907^o^ S and Cardwell, 18.267^o^ S) populations [18, 22]. The temperate and tropical populations show extensive genomic variation, suggesting spatially varying selection of numerous biological processes [22]. We obtained 8 independent isofemale lines for each region, and data were pooled to derive group averages for temperate and tropical populations. Males and females were separately analyzed because there is sexual dimorphism in mid-day sleep levels whereby females generally sleep less during the day [25, 26]. As discussed below, 26 additional lines were subsequently analyzed in a more limited study to strengthen the results obtained with the original 16 lines.

To measure daily sleep levels in *Drosophila* we used the standard approach of counting beam-crossings whereby sleep is routinely defined as no detection of locomotor activity movement for any period of at least 5 contiguous min [25]. Young adult flies were entrained (synchronized) under standard laboratory conditions of 12 hr light/12 hr dark cycles [LD; where zeitgeber time (ZT) 0 is lights-on] at different temperatures (18^o^, 25^o^ and 29°C), and then maintained for a week under constant dark conditions (DD) to measure free-running activity rhythms [5, 27]. The majority of sleep occurs during the night in *D*. *melanogaster* but some sleep is also observed during the day [25, 28]. Sleep in *D*. *melanogaster* is characterized by closely spaced sleep episodes that vary in length and number. Daytime sleep is usually limited to the middle of the day and is more fragmented (e.g., shorter sleep bout durations) compared to sleep at night [28].

**Fig 1:**
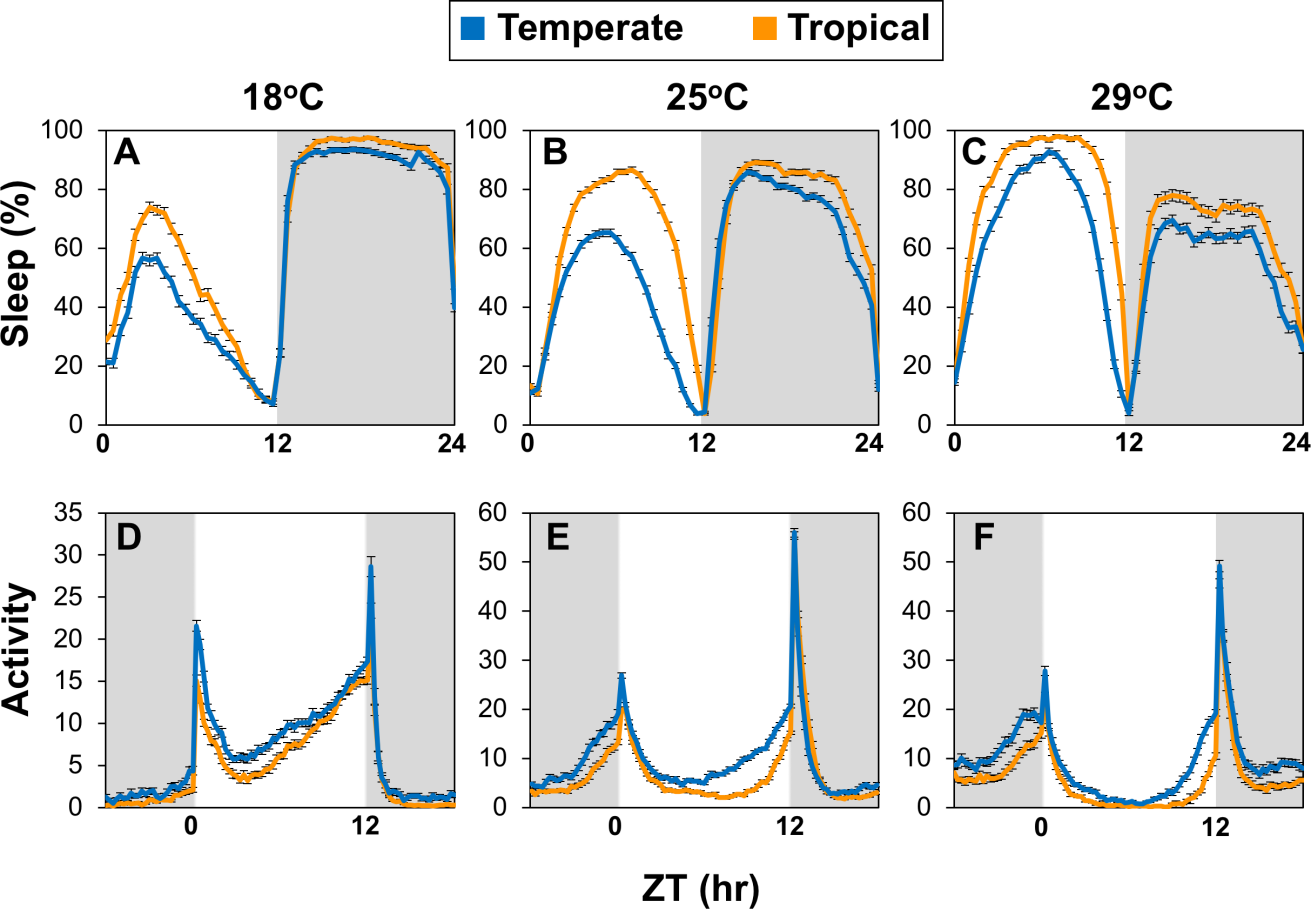
Australian flies from temperate regions exhibit less sleep during the day compared to tropical populations. (A-F) Adult male flies from 8 different tropical isofemale lines and 8 different temperate isofemale lines were kept at the indicated temperature (top of panels) and entrained for five days of 12 hr light/12 hr dark cycles [LD; where zeitgeber time 0 (ZT0) is lights-on]. For each isofemale line, the locomotor activity of individual flies (n = 16 for 18^o^ and 29°C; n = 32 for 25°C) was measured, followed by pooling the data to obtain a group average for the tropical and temperate populations. The last three days’ worth of LD data was pooled, and shown are the daily sleep levels in 30 min bins. Fly lines used are as follows; tropical, HB22, HB25, HB27, HB106, HB108, GT46, GT92, GT110; temperate, S3, S4, S7, S8, S12, S22, S28, S34. (A-C) Shown are group averages for daily sleep (expressed as the percentage of time flies were sleeping during 30 min time-windows). (D-F) Shown are group averages for daily activity rhythms. To facilitate comparisons of daily activity profiles, the peak value in daily activity for each fly was set to 1.0 and the normalized profiles superimposed. ZT, zeitgeber time (hr). The gray shading in the panels represents dark periods. Tropical populations exhibit increased daytime sleep, which was also observed in female flies (see S1 Fig). The following *p* values were determined: [one-sided *t-test*; panel A, daytime values (ZT0-12), *p* = 7 x 10^−4^; for nighttime values (ZT12-24), *p* = 0.058], [panel B, ZT0-12, *p* < 0.00001; ZT12-24, *p* = 0.011], [panel C, ZT0-12, *p* < 0.00001; ZT12-24, *p* = 0.0006].

**Fig 2:**
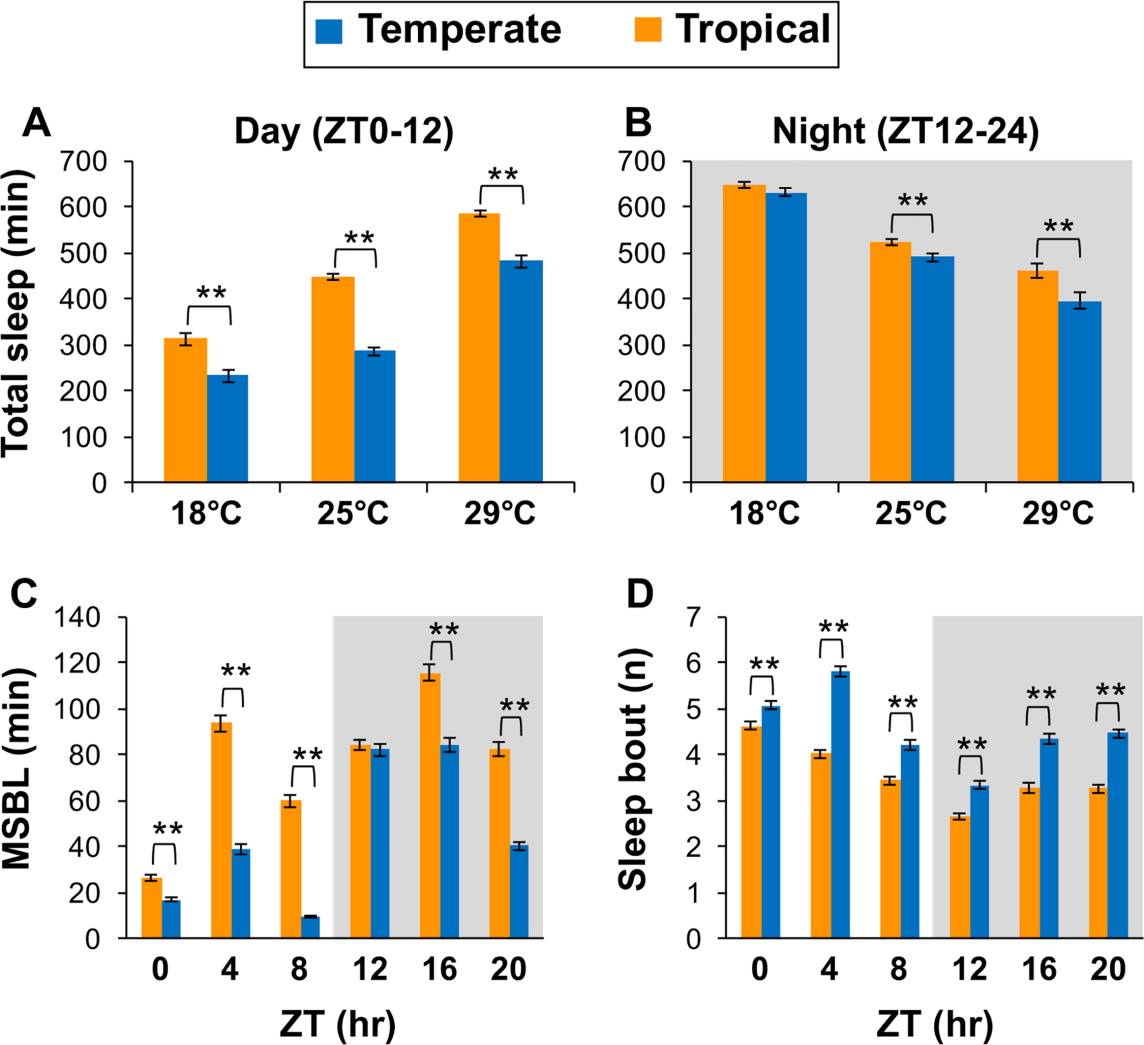
Daytime and nighttime sleep is more fragmented in temperate flies. (A-D) The results are based on the same flies and activity data used in Fig 1. (A, B) Shown are group averages for total amount of sleep during 12 hr of day (A) and 12 hr of night (B), averaged over the last three days of LD. (C, D) Shown are group averages for median sleep bout length (MSBL) (C) and number of sleep bouts (D), during 4 hr time-windows. Values for tropical and temperate populations are significantly different using one-sided Student’s *t-test*; *, p < 0.05; **, p < 0.01. The following *p* values were determined; [panel A; 18°C, 0.0018; 25°C, <0.001; 29°C, <0.001], [panel B; 18°C, 0.33; 25°C, 0.0087; 29°C, 0.0017], [panel C; ZT0, 0.0011; ZT4, <0.001; ZT8, <0.001; ZT12, 0.38; ZT16, <0.001; ZT20, <0.001], [panel D; ZT0, 0.0031; ZT4, <0.001; ZT8, <0.001; ZT12, <0.001; ZT16, <0.001; ZT20, <0.001].

At all temperatures tested, temperate populations manifest less sleep during the day compared to tropical populations, whereas nighttime sleep levels showed less latitudinal differences (Fig 1A–1C; Fig 2A and 2B). Although we mainly analyzed sleep behavior in males, both sexes showed similar latitudinal differences in mid-day sleep levels (S1 Fig). As previously observed, increases in temperature lead to progressively higher total daytime sleep levels and conversely, larger reductions in nighttime sleep (Fig 2A and 2B) [11, 29]. This is presumably because increased sleep during the day attenuates the accumulation of sleep debt during the night and/or night sleep is more disrupted by heat [29]. Because both tropical and temperate flies exhibit a gradual increase in mid-day siesta as temperature increases, this suggests that their thermal sensing systems for sleep are functional but differentially calibrated. Analysis of sleep behavior showed that the decreased daytime sleep in temperate flies is attributable to significant decreases in the average length of sleep episodes (MSBL) and an increase in the number of sleep episodes (Fig 2C and 2D). Although total nighttime sleep showed less differences in total levels compared to daytime sleep (Fig 2B), it was less consolidated in temperate flies as evidenced by shorter and more frequent sleep episodes (Fig 2C and 2D). The overall decreased and more fragmented sleep phenotypes observed in temperate compared to tropical populations are not due to hyper-activity as all flies showed similar activity levels during wake periods (S2 Fig).

These findings are remarkably similar to those comparing natural populations of *D*. *melanogaster* from low and high altitudes in tropical Africa [12]. For example, African flies adapted to high altitudes exhibit significantly less daytime sleep levels that are characterized by an increase in sleep episodes of reduced length, and while nighttime sleep shows little altitudinal changes in total levels, it is less consolidated. The fact that both high altitude African flies and those from temperate regions in Australia show parallel clinal changes in sleep behavior further supports the claim that mid-day sleep in these natural populations is a key thermal adaptation to varying local climates [21].

In addition to sleep, we also measured the daily activity rhythm. In LD cycles, the daily distribution of activity in *D*. *melanogaster* exhibits two clock-controlled bouts of activity (‘morning’ and ‘evening’) that are separated by a mid-day dip in activity or siesta [6, 7]. Overlaying the clock regulation of daily activity are more acute responses caused by the light/dark transitions that evoke transient increases in activity (sometimes referred to as ‘startle’ responses or masking effects) [5, 6] (e.g., Fig 1D–1F). As expected from the sleep analysis, tropical flies were preferentially less active during the day compared to temperate flies, although all showed the typical delay in the onset of evening activity and lower daytime activity with increased temperature [7].

To analyze circadian properties of the daily wake-sleep cycles, activity data was recorded for a week in DD following entrainment to LD. Both the tropical and temperate populations exhibit robust free-running behavioral rhythms of approximately 24 hr over a broad range of temperatures (S1 Table). This is a hallmark feature of circadian rhythms termed temperature compensation, wherein period length remains relatively constant over a wide range of physiologically relevant temperatures [30]. Because behavioral periods are similar in the temperate and tropical populations, it is unlikely that the geographical differences observed in daily sleep levels are due to alterations in the pace of the circadian clock. Therefore, the differences in the daily sleep patterns of the Australian tropical and temperate populations are reminiscent of the behavioral effects of dmpi8 splicing which preferentially modulates daytime sleep levels without evoking changes in clock speed [7, 8].

### Geographical differences in sleep continue in constant light conditions, revealing a strong non-circadian component

**Fig 3:**
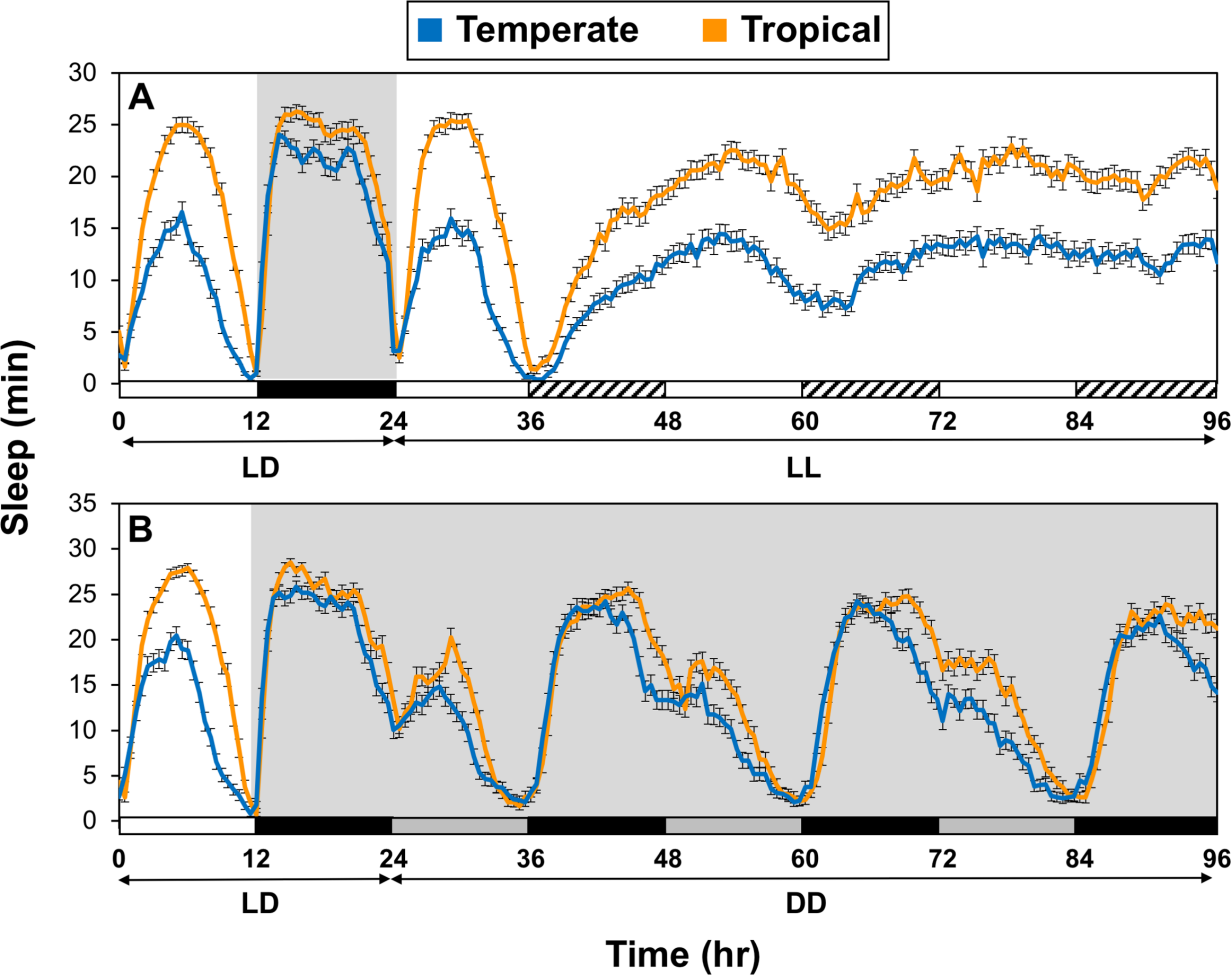
Reduced sleep in temperate flies continues in constant light conditions where circadian clock function is abolished. (A, B) Shown are group averages of sleep levels in min during 30-min bins. Young adult male flies were maintained at 25°C and entrained for five days in LD. Subsequently, half of the tropical and temperate groups were placed in constant light (LL; panel A), whereas the other half was placed in constant darkness (DD; panel B). Time 0 (hr) begins with the fifth day of LD followed by the subsequent days in LL or DD. For the data shown in this figure, 16 individual flies from each of the 8 lines representing each region (tropical and temperate) were analyzed and data pooled. Fly lines used are as follows; tropical, HB22, HB25, HB27, HB106, HB108, GT46, GT92, GT110; temperate, S3, S4, S7, S8, S12, S22, S28, S34. White, black, stripped, and light gray horizontal bars below panels represent 12-hr periods of light, dark, ‘subjective nighttime in LL, and ‘subjective daytime’ in DD, respectively. In LL and DD, comparison of tropical and temperate flies for daily sleep levels showed highly significant differences (one-way ANOVA for first 3 days in LL or DD; panel A, p<0.0001; panel B, 0.0091).

In recent work using transgenic flies that were engineered to have different dmpi8 splicing efficiencies, we showed that compared to control wildtype flies (termed 8:8), flies with higher dmpi8 splicing efficiency (termed M2M1) exhibit less sleep even during extended periods of constant light (LL), which conditionally abolishes clock function [11]. In addition, flies from high altitudes in Africa also manifest less sleep in extended LL compared to low altitude flies [12]. Likewise, *D*. *melanogaster* from the tropical locations in Australia have higher sleep levels in LL compared to the temperate populations (Fig 3A). Thus, although tropical-temperate differences in the daily wake/sleep patterns continue to some degree in constant darkness (Fig 3B), a functional clock is not required.

### The splicing efficiency of dmpi8 correlates with the tropical-temperate differences observed in mid-day sleep levels

**Fig 4:**
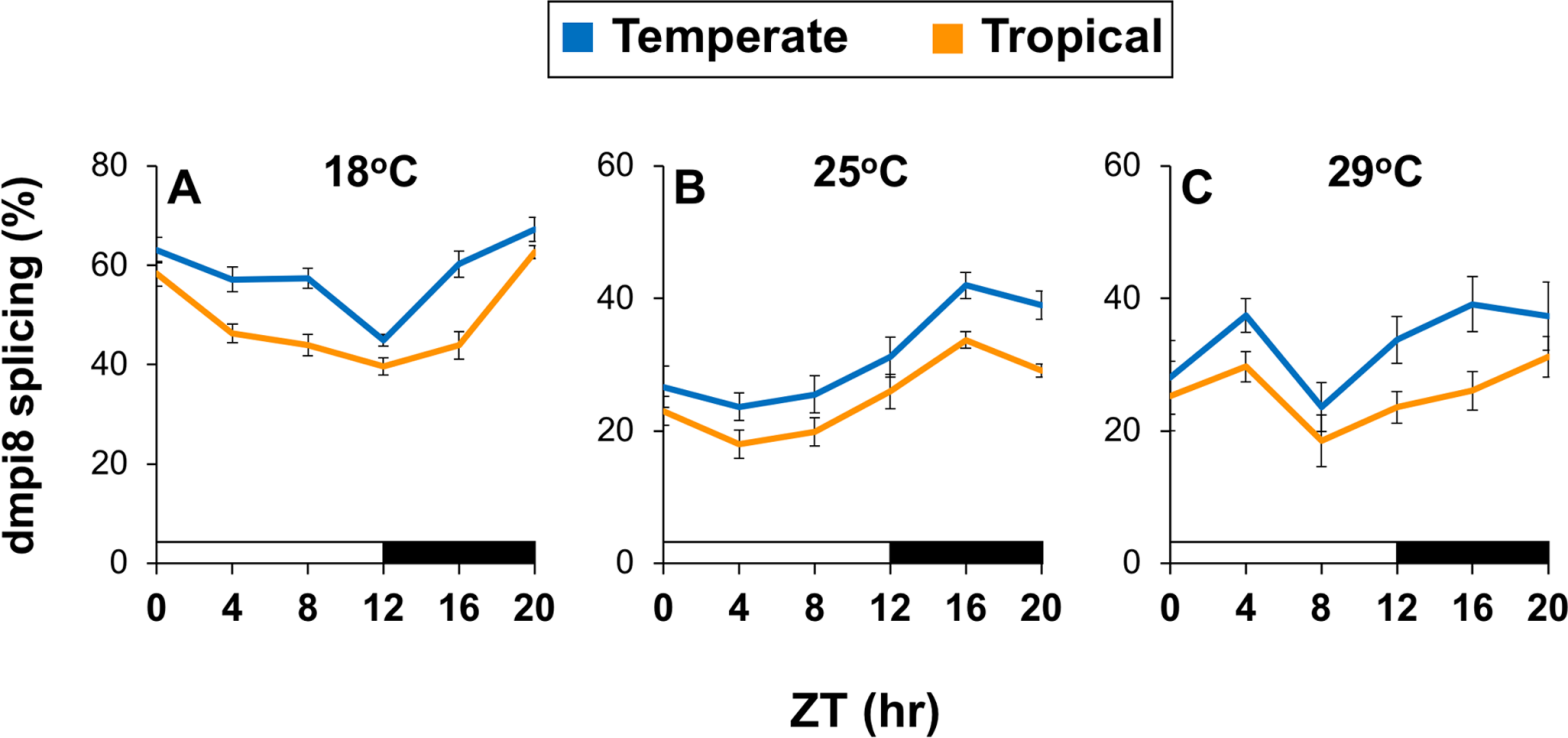
Daily splicing efficiency of the dmpi8 intron is higher in temperate flies compared to tropical flies. (A-C) Shown are group averages for the splicing efficiency of dmpi8 (expressed as the proportion of *dper* transcripts where the dmpi8 intron was spliced) throughout an LD cycle (where ZT0 is lights-on) for tropical and temperate flies. The 16 different lines of Australian flies were entrained for five days of LD at the indicated temperatures (top of panels), and flies from individual lines collected separately on the fifth day by freezing at the indicated times (ZT, hr). Extracts were prepared from isolated heads and dmpi8 splicing efficiency measured for each line separately, followed by pooling results from different lines to yield group averages for tropical and temperate populations. Fly lines used are as follows; tropical, HB22, HB25, HB27, HB106, HB108, GT46, GT92, GT110; temperate, S3, S4, S7, S8, S12, S22, S28, S34. For each temperature, the daily dmpi8 splicing curves were significantly different between the tropical and temperate populations (one-way ANOVA; 18°C, *p* = 1.3 x10^-5^; 25°C, *p* = 0.0021; 29°C, *p* = 6.8 x 10^−4^). Similar results were obtained in two independent experiments and a representative example is shown.

To measure dmpi8 splicing efficiency, flies were kept at the three different test temperatures (i.e., 18^o^, 25^o^ and 29°C), collected at different times throughout a daily cycle and dmpi8 splicing measured in head extracts. The daily splicing efficiency of dmpi8 manifests a low amplitude rhythm and shows overall increases in levels as temperature decreases [7, 31, 32]. Based on prior work we predicted that if dmpi8 splicing efficiency contributes to the differences in the daytime sleep behavior observed in the temperate and tropical populations, then it should have higher overall daily levels in temperate flies [7]. Indeed, at all temperatures tested the daily splicing efficiency of the dmpi8 intron is higher in Australian flies from temperate regions compared to their tropical counterparts (Fig 4). The general shapes of the daily dmpi8 splicing curves for tropical and temperate flies were similar at the same temperature, suggesting that latitude affects the splicing efficiency of dmpi8 in a largely circadian independent manner (note; the pattern observed at 29°C is somewhat atypical, as usually only one peak is observed for dmpi8 daily splicing efficiency). We also measured dmpi8 intron removal during the first day of DD (DD1) and during the third day of LL (LL3) and observed a similar trend whereby the overall daily splicing efficiency is higher in temperate flies (S3 Fig). Thus, there is a strong correlation between the splicing efficiency of the dmpi8 intron and daytime sleep levels in the temperate and tropical populations.

### Australian flies contain two major haplotypes of the *dper* 3’ UTR that differ in relative frequency between the tropical and temperate populations

**Fig 5:**
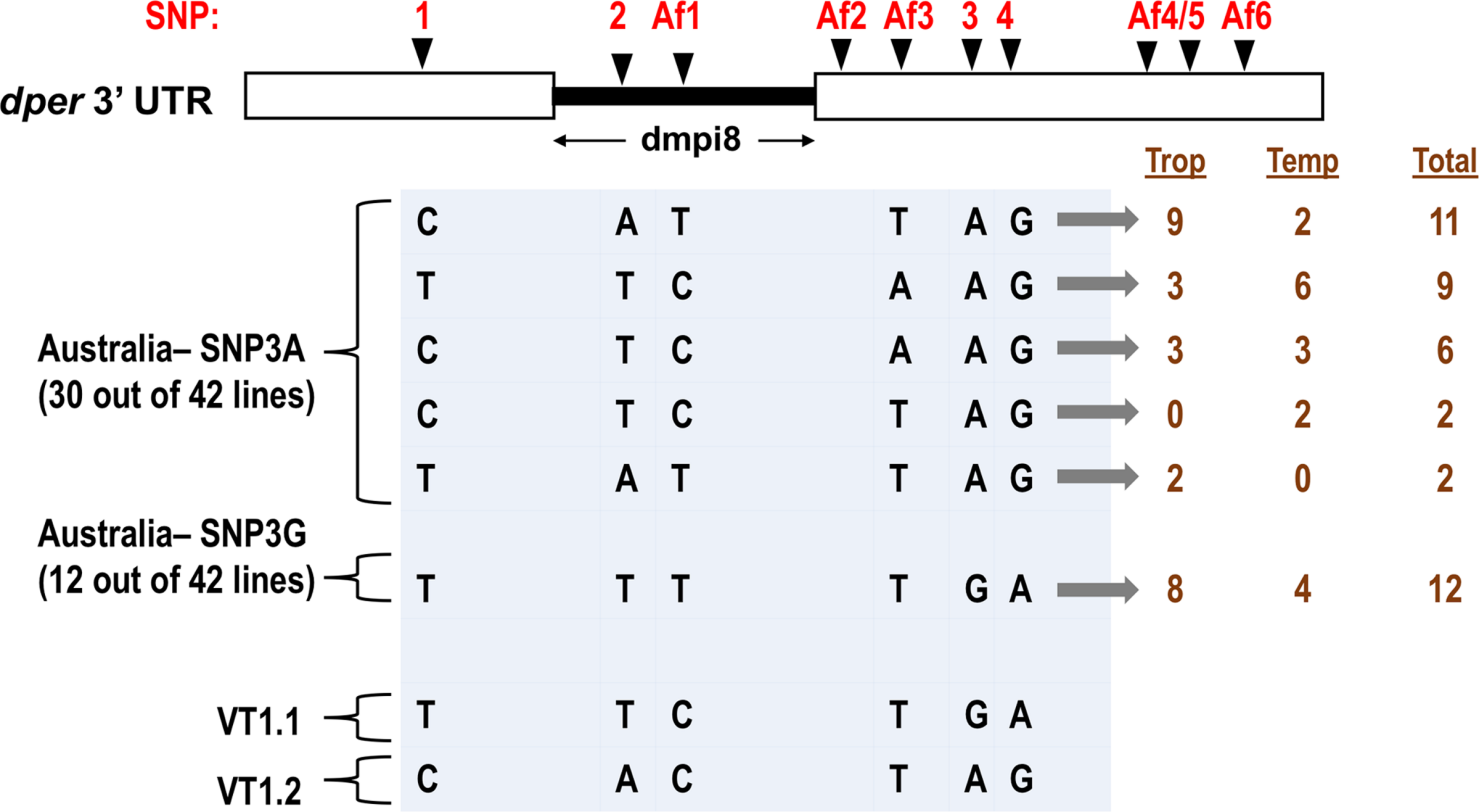
Reciprocal tropical-temperate distribution pattern for two major *dper* 3’ UTR haplotypes found in Australian flies. At the top is shown a schematic of the *dper* 3’ UTR with the different SNPs identified to date based on sequencing natural populations from tropical Africa, the eastern coast of the United States and Australia. The SNPs termed 1–4 are based on the original description of flies from the United States [16]. Those termed Af1-Af6 are based on more recent work analyzing flies from tropical Africa [12] and the Australian flies analyzed in this study (see S1 Appendix). Only those SNPs that differ in the Australian populations analyzed in this study are listed below (i.e., SNPs1-4, Af1 and Af3; light blue shading). For flies from Australia, 30 populations have SNP3A, whereas 12 populations have SNP3G. For each different *dper* 3’ UTR haplotype identified, the SNP variants at positions SNPs1-4, Af1 and Af3 are shown. At the right is shown the number of tropical (trop), temperate (temp) and total fly populations for each *dper* 3’ UTR haplotype from Australia (dark brown). At the bottom are shown the VT1.1 and VT1.2 variants previously reported in [16]. The C/A/T/T and T/T/C/A containing populations showed significantly different relative spatial distributions (*Chi-*square test, *p* = 0.027; Fisher exact test, *p* = 0.039).

We previously reported the identity of four major single nucleotide polymorphisms (SNPs; termed SNPs 1–4) in the *dper* 3’ untranslated region (UTR) from natural populations of *D*. *melanogaster* that were originally caught along the eastern coast of the United States [16, 33] (see Fig 5, bottom). These four SNPs segregated into two main *dper* 3’ UTR haplotypes termed VT1.1 and VT1.2, wherein the VT1.1 version exhibits higher dmpi8 splicing efficiency and reduced mid-day siesta [16]. We also noted two small deletions/additions that differ between VT1.1 and VT1.2, but evidence indicates that they have little to no influence on daytime sleep levels or dmpi8 splicing [16]. More recent work using natural populations of *D*. *melanogaster* from different altitudes in equatorial Africa [12] revealed the same SNPs as those found in the United States, in addition to other SNPs (Fig 5, top). Our studies using flies from the United States and Africa identified a particular SNP (termed SNP3) that has strong effects on dmpi8 splicing efficiency and mid-day sleep [12, 16]. SNP3 has two variants, G or A, and evidence to date indicates that when SNP3 is a G (as is found in VT1.1) it has dominant effects irrespective of other SNPs in the *dper* 3’ UTR, leading to increased dmpi8 splicing and reduced mid-day sleep [12, 16].

**Fig 6:**
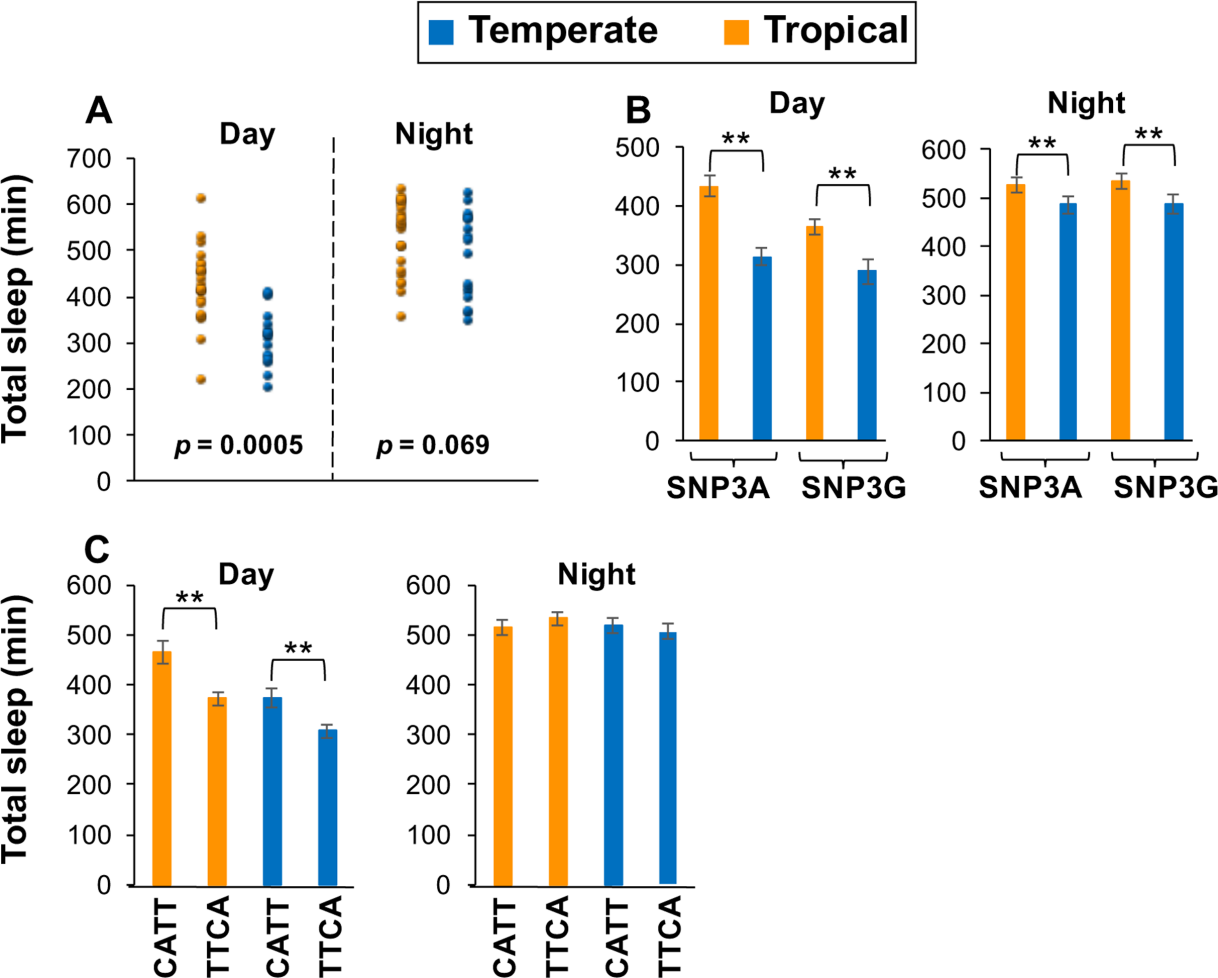
Natural populations of flies that carry the major temperate haplotype (T/T/C/A) manifest decreased daytime sleep compared to the major tropical haplotype (C/A/T/T). (A-C) Adult male flies from the 42 different populations analyzed in this study (see S1 Appendix and S5 Fig) were kept at 25°C and entrained for five days of LD. For each isofemale line, the locomotor activity of individual flies (n = 16) was measured and data from the last three days of LD pooled to obtain values for the total amount of sleep during the 12 hr of day and the 12 hr of night. Data from the different populations was pooled in different ways to generate the following temperate-tropical comparisons; (A) all 42 populations, (B) SNP3A (30 populations) versus SNP3G (12 populations), and (C) C/A/T/T (11 populations) versus T/T/C/A (9 populations). **, p < 0.01. The following *p* values were obtained using one-sided Student’s *t-test*; [panel A; temperate vs tropical; day, *p* = 0.0005; night, *p* = 0.069], [panel B, temperate vs tropical during the day; SNP3A, *p* <0.0001; SNP3G, *p* <0.0001], [panel B, temperate vs tropical during the night; SNP3A, *p* <0.0001; SNP3G, *p* <0.0001], [panel C; tropical C/A/T/T vs tropical T/T/C/A during the day; *p* <0.0001; temperate C/A/T/T vs temperate T/T/C/A during the day; *p* = 0.0004], [panel C; tropical C/A/T/T vs tropical T/T/C/A during the night; *p* = 0.36; temperate C/A/T/T vs temperate T/T/C/A during the night; *p* = 0.28].

To determine if SNPs in the *dper* 3’ UTR might contribute to differences in daytime sleep levels between the temperate and tropical Australian populations, we sequenced the *dper* 3’ UTR region for each of the 16 Australian *D*. *melanogaster* populations described above. In addition, to bolster the sequencing analysis we were able to obtain an additional 26 independent tropical and temperate populations from the same well-studied Australian *D*. *melanogaster* cline analyzed above [18, 22] (Fig 5; S1 Appendix). A smaller scale behavioral study of the 26 additional lines showed that flies from the tropical region have significantly increased daytime sleep compared to their temperate counterparts (S4 and S5 Figs). When data from all 42 populations were combined, it is clear that as a group temperate flies manifest significantly less daytime sleep compared to tropical populations, whereas nighttime sleep levels show less geographical variation (Fig 6A and S5 Fig). Thus, the combined results from the 42 different populations (26 tropical, 17 temperate) strongly suggest that adaptation of *D*. *melanogaster* along the Australian cline involved spatial selection for mid-day siesta levels that are more optimized to local temperature ranges (see Discussion).

Sequencing of the *dper* 3’ UTR from the 42 Australian lines identified numerous SNPs that are organized into 6 different haplotypes (Fig 5, light blue shading). The sequences are listed as supplementary information (S1 Appendix). To maintain consistency with prior published work, the 4 SNPs we previously reported in flies from the United States are referred to by their original designation (SNP1-4) [16], whereas those more recently identified in African populations are herein designated Africa (Af) 1–6 (Fig 5, top). For the purposes of this report only those SNPs that we observed in flies from Australia are detailed (Fig 5; S1 Appendix). A more comprehensive description and analysis of *dper* 3’ UTR sequences and SNPs from other regions (e.g., Africa) will be presented elsewhere (manuscript in preparation). For flies from Australia, the main variants include SNPs 1–4 and two that are also prevalent in Africa, namely Af1 and Af3 (Fig 5, top). It should be noted that Af1 and Af3 were also observed in flies from the eastern coast of the United States [16] but we did not report these SNPs in the original characterization of these fly populations as they were extremely rare. Similar to SNPs1-4 and other SNPs that are more prevalent in the *dper* 3’ UTRs from African flies, SNPs Af1 and Af3 only have two alleles each (Fig 5). It therefore appears that all the current SNPs in the *dper* 3’ UTRs found in the more cosmopolitan strains from the United States or Australia were already present in ancestral African populations (see Discussion). This is not surprising given that African populations exhibit the most genetic diversity [15].

Of the 42 independent Australian populations, 12 have SNP3G and the rest are SNP3A (Fig 5, right; see S1 Appendix). When behavioral data from all the SNP3A flies were pooled, temperate populations showed lower daytime sleep levels compared to the tropical populations (Fig 6B, left panel). A similar temperate-tropical trend was also observed with nighttime sleep levels, although the magnitude of the difference was smaller compared to daytime values (Fig 6B, right panel). Likewise, SNP3G flies from temperate regions also showed decreased daytime sleep levels compared to their tropical counterparts (Fig 6B, left panel). Consistent with prior work, SNP3G flies manifest less daytime sleep compared to SNP3A flies from the same region, especially for tropical populations (Fig 6B, left panel) [12, 16]. Irrespective of location, all the SNP3G-containing flies have the exact same *dper* 3’ UTR sequence (Fig 5 and S1 Appendix), indicating that any tropical-temperate differences in the mid-day sleep levels of SNP3G flies (Fig 6B) is not based on spatial variations in *dper* 3’ UTR SNPs.

In sharp contrast to SNP3G flies, the SNP3A-containing flies yielded 5 haplotypes that differ in combinations of SNPs 1, 2, Af1 and Af3 (Fig 5). Intriguingly, for the two most abundant SNP3A haplotypes (20 out of 30; Fig 5, right), they showed an inverted geographical distribution in their relative frequencies (Fig 5). The main combination of SNP1/SNP2/Af1/Af3 that we noticed for tropical populations was C/A/T/T (herein also designated p3’AusTrop), whereas for temperate populations it was T/T/C/A (herein also designated p3’AusTemp). In tropical regions, 9 populations have C/A/T/T, whereas only 3 have T/T/C/A. Conversely, in temperate regions, 6 populations have the T/T/C/A but only 2 have C/A/T/T. This raises the interesting possibility that the spatial distributions of the C/A/T/T and T/T/C/A variants are somehow dynamically linked. *Chi*-square analysis showed a significant difference in the relative distributions of the C/A/T/T and T/T/C/A variants (*p* = 0.027). The other less prominent SNP3A haplotypes either did not show spatial variation or were too minor to draw any solid conclusions (Fig 5).

Remarkably, for each region (i.e., temperate or tropical), fly populations carrying the major tropical variant (C/A/T/T) sleep significantly more during the day compared to populations carrying the major temperate variant (T/T/C/A) (Fig 6C, left). Nighttime sleep levels were similar irrespective of haplotype or geographical region (Fig 6C, right). These results strongly suggest that the *dper* 3’ UTR haplotype plays a prominent role in setting the daytime sleep levels of natural populations carrying the C/A/T/T and T/T/C/A variants. Besides the type of *dper* 3’ UTR haplotype, other factors almost certainly contribute to setting daytime sleep levels in these populations because for a given haplotype (e.g., tropical C/A/T/T versus temperate C/A/T/T), flies from tropical regions exhibit higher daytime sleep levels compared to their temperate counterparts (Fig 6C, left).

In summary, we identified two major *dper* 3’ UTR haplotypes that comprise approximately 50% of the variants found in 42 natural populations of *D*. *melanogaster* from the east coast of Australia (Fig 5). For a given region, those with the C/A/T/T variant exhibit significantly increased daytime sleep compared to those carrying the T/T/C/A variant (Fig 6C). The largest tropical-temperate differences in daytime sleep levels for these two haplotypes are tropical C/A/T/T compared to temperate T/T/C/A flies (Fig 6C, left). Sequencing studies reveal a reciprocal tropical-temperate distribution pattern for these two major haplotypes in Australia (Fig 5). Although spatial variation in *dper* 3’ UTR haplotypes cannot explain all the temperate-tropical differences in daytime sleep levels, it follows from the combined findings that the inverted spatial distribution of two major haplotypes with significantly different mid-day sleep levels should have a strong impact on the overall maintenance of a robust cline in the daytime sleep levels of Australian flies. Because the C/A/T/T and T/T/C/A variants 1) have not been studied before, 2) are associated with sleep differences in natural populations that follow the overall tropical-temperate cline, 3) are major variants found in Australia, and 4) are the first example of possible spatial selection in *dper* 3’ UTR haplotypes, we focused on these two haplotypes and sought to obtain more direct evidence that they regulate dmpi8 splicing efficiency and mid-day sleep levels.

### A simplified cell culture assay reveals differences in the splicing efficiency of dmpi8 for the major tropical and temperate SNP combinations

**Fig 7:**
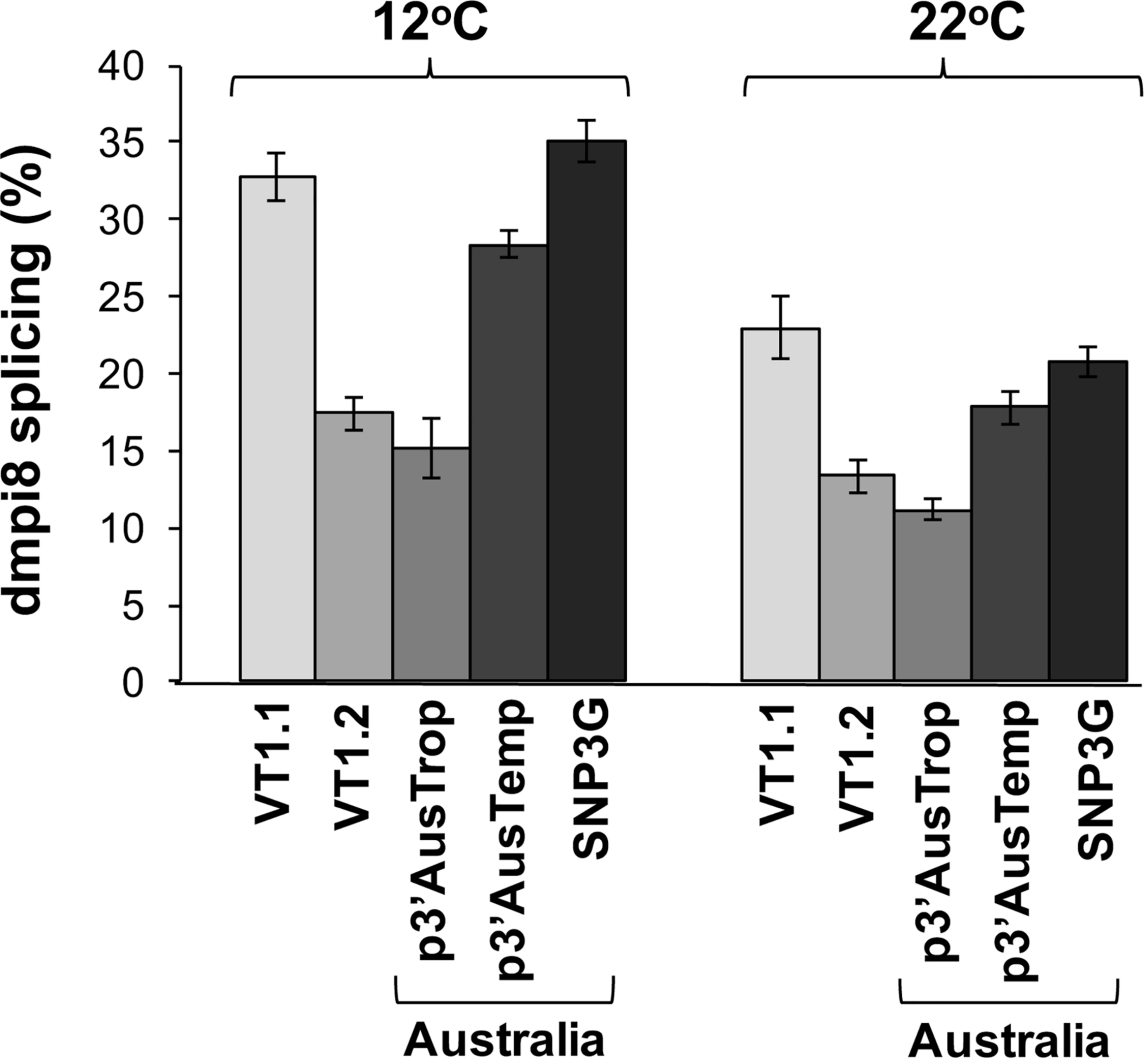
The major tropical and temperate sequences for the *dper* 3’ UTR exhibit differential dmpi8 splicing efficiency in *Drosophila* S2 cells. *Drosophila* S2 cells were transfected with the indicated plasmids (bottom of panels), grown at either 12^o^ or 22°C (as indicated, top), and the splicing efficiency dmpi8 calculated (shown as the percentage of transcripts where the dmpi8 intron is spliced). p3’AusTrop, refers to the major tropical *dper* 3’ UTR haplotype (C/A/T/T), whereas p3’AusTemp refers to the major temperate one (T/T/C/A). SNP3G refers to the *dper* 3’ UTR found in the Australian flies (see Fig 5 and S1 Appendix). The results shown are based on an average of 5 independent experiments. The following p values were determined (one-sided Student’s *t*-test): [12°C; VT1.1 vs VT1.2, *p* = 2.3 x 10^−5^; VT1.1 vs SNP3G, *p* = 0.24; VT1.1 vs p3’AusTrop, *p* = 1.2 x 10^−9^; VT1.1 vs p3’AusTemp, *p* = 0.17; p3’AusTemp vs p3’AusTrop, p = 1.8 x 10^−5^; SNP3G vs p3’AusTrop, p = 4.0 x 10^−10^; SNP3G vs p3’AusTemp, *p* = 0.07; p3’AusTrop vs VT1.2, *p* = 0.11; p3’AusTemp vs VT1.2, *p* = 0.01], [22°C; VT1.1 vs VT1.2, *p* = 0.0012; VT1.1 vs SNP3G, *p* = 0.22; VT1.1 vs p3’AusTrop, *p* = 3.5 x 10^−9^; VT1.1 vs p3’AusTemp, *p* = 0.0043; p3’AusTemp vs p3’AusTrop, *p* = 7.9 x 10^−8^; SNP3G vs p3’AusTrop, *p* = 2.2 x 10^−8^; SNP3G vs p3’AusTemp, *p* = 0.069; p3’AusTrop vs VT1.2, *p* = 0.019; p3’AusTemp vs VT1.2, *p* = 0.008].

As an initial attempt to test if the splicing efficiency of the dmpi8 intron differs between the two main SNP combinations (i.e., T/T/C/A and C/A/T/T) we used a simplified *Drosophila* S2 cell culture assay that we previously showed recapitulates the temperature dependent splicing of dmpi8 [8] (Fig 7). In addition, we also included the Australian SNP3G haplotype (Fig 5 and S1 Appendix) and the VT1.1 and VT1.2 haplotypes originally identified in populations of flies from the eastern coast of the United States [16] (the other more minor variants found in Australia were not analyzed in this study). In our cell culture assay the *dper* 3’ UTR and approximately 90bp of 3’ non-transcribed DNA is subcloned downstream of the luciferase open reading frame (*luc*) [8, 16]. After transfection, S2 cells were grown at either 12^o^ or 22°C and dmpi8 splicing efficiency measured. As expected, of the three Australian variants tested, the SNP3G-containing variant exhibited the highest dmpi8 splicing efficiency, reaching levels very similar to that observed for VT1.1, further confirming that when SNP3G is present in the *dper* 3’ UTR, dmpi8 splicing is enhanced to similar levels irrespective of other SNPs (Fig 7). Remarkably, for SNP3A-containing *dper* 3’ UTRs, splicing of dmpi8 is significantly higher in the p3’AusTemp background compared to p3’AusTrop (Fig 7). The splicing efficiency of dmpi8 in the major temperate variant (p3’AusTemp) is less than that of those containing SNP3G (i.e., VT1.1 and SNP3G), whereas the major tropical sequence (p3’AusTrop) appears similar to, or slightly less, then VT1.2. Thus, the enhanced splicing efficiency of the major temperate variant (T/T/C/A) compared to the major tropical variant (C/A/T/T) is consistent with the daytime sleep profiles observed in natural populations (Fig 6C).

### Transgenic models directly show that flies with the T/T/C/A haplotype have decreased siesta and enhanced dmpi8 splicing efficiency compared to those with C/A/T/T

Encouraged by results obtained in the simplified cell culture system, we sought to evaluate the physiological significance of the p3’AusTemp (T/T/C/A) and p3’AusTrop (C/A/T/T) haplotypes by generating transgenic flies that have identical genetic backgrounds except that they differ in the type of *dper* 3’ UTR, herein termed p{p3’AusTemp} and p{p3’AusTrop}, respectively. In addition, we also generated transgenic flies carrying the SNP3G-containing variant from Australia (p{3’AusSNP3G}). The germ-line transformants were crossed into a *wper*^0^ genetic background so that the only functional copy of *dper* is the transgene (e.g., [16]). Numerous independent lines were obtained and analyzed for each genotype.

**Fig 8:**
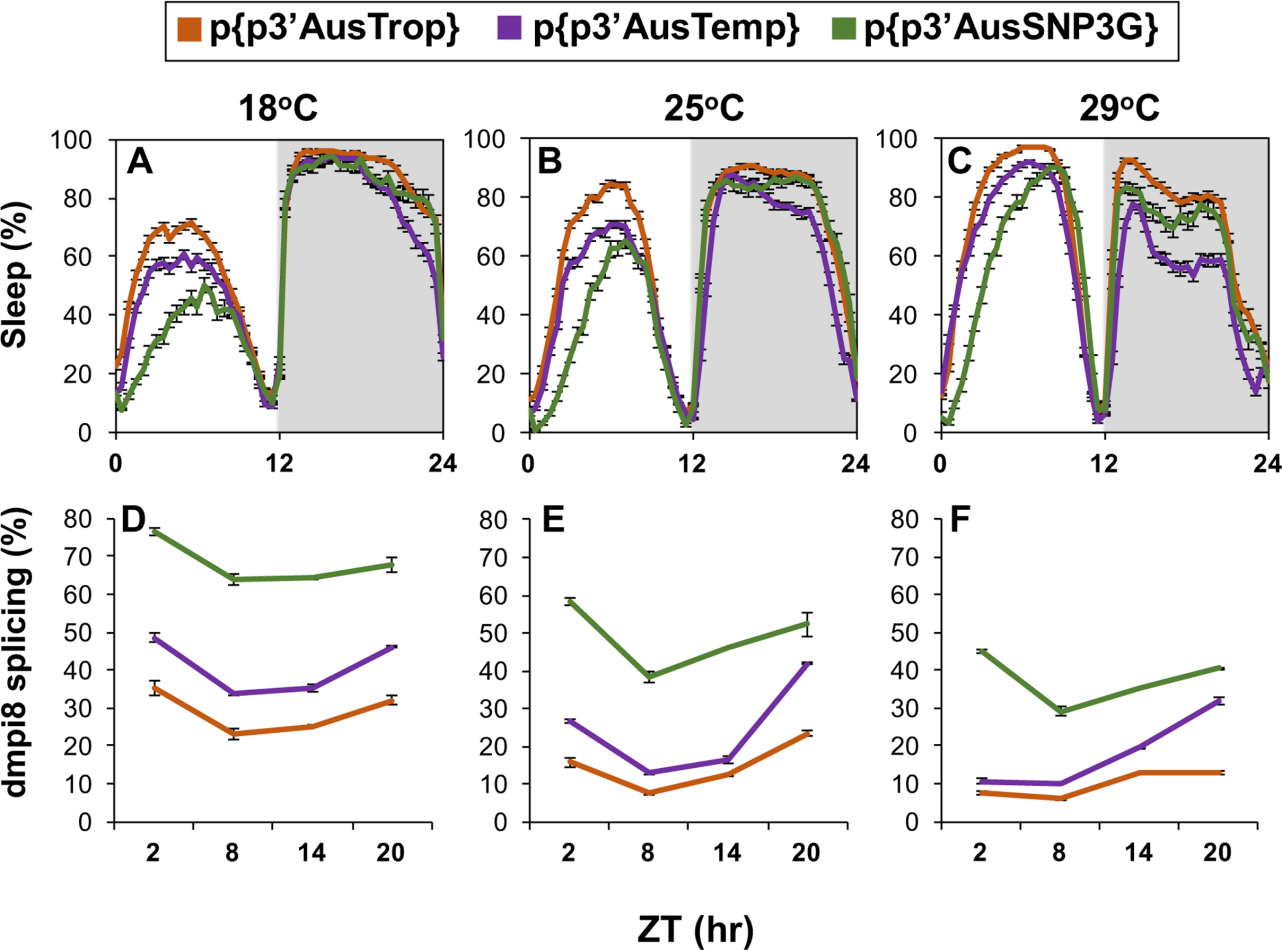
Analysis of mid-day siesta and dmpi8 splicing in transgenic flies carrying the major temperate and tropical *dper* 3’ UTR haplotypes. (A-C) Shown are group averages for daily sleep (expressed as the percentage of time flies were sleeping during 30-min time-windows). Male flies of the indicated genotype were entrained for five cycles of 12 hr light/12 hr dark cycles (LD; where ZT0 is lights-on) at the indicated temperature (top of panels), and the last three LD worth of data used to generate the profiles shown. p{p3’AusTrop}, transgenic flies carrying the major tropical *dper* 3’ UTR; p{p3’AusTemp}, transgenic flies carrying the major temperate *dper* 3’ UTR; and p{p3’AusSNP3G}, transgenic flies carrying the SNP3G variant observed in Australia. For transgenic flies, at least three independent lines for each construct were analyzed (16 flies per line) and data pooled [3 lines for p{p3’AusSNP3G}; 4 lines for p{p3’AusTemp}; and 6 lines for p{p3’AusTrop}]. Pair-wise comparisons showed that the different genotypes showed significant differences in daytime sleep levels from each other (one-sided Student’s *t-test*). [For 18°C, daytime sleep values (ZT0-12); p{p3’AusTemp} vs p{p3’AusTrop}, *p* = 5.0 x 10^−4^; p{p3’AusTemp} vs p{p3’AusSNP3G}, *p* = 0.0017; p{p3’AusTrop} vs p{p3’AusSNP3G}, *p* < 1.0 x 10^−5^], [For 18°C, nighttime sleep values (ZT12-24); p{p3’AusTemp} vs p{p3’AusTrop}, *p* < 1.0 x 10^−5^; p{p3’AusTemp} vs p{p3’AusSNP3G}, *p* = 0.074; p{p3’AusTrop} vs p{p3’AusSNP3G}, *p* = 0.045], [For 25°C, daytime sleep values (ZT0-12); p{p3’AusTemp} vs p{p3’AusTrop}, *p* = 4.0 x 10^−4^; p{p3’AusTemp} vs p{p3’AusSNP3G}, *p* = 0.0024; p{p3’AusTrop} vs p{p3’AusSNP3G}, *p* < 1.0 x 10^−5^], [For 25°C, nighttime sleep values (ZT12-24); p{p3’AusTemp} vs p{p3’AusTrop}, *p* < 1.0 x 10^−5^; p{p3’AusTemp} vs p{p3’AusSNP3G}, *p* = 0.0033; p{p3’AusTrop} vs p{p3’AusSNP3G}, *p* = 0.34], [For 29°C, daytime sleep values (ZT0-12); p{p3’AusTemp} vs p{p3’AusTrop}, *p* = 4.0 x 10^−4^; p{p3’AusTemp} vs p{p3’AusSNP3G}, *p* = 0.01; p{p3’AusTrop} vs p{p3’AusSNP3G}, *p* < 1.0 x 10^−5^], [For 29°C, nighttime sleep values (ZT12-24); p{p3’AusTemp} vs p{p3’AusTrop}, *p* < 1.0 x 10^−5^; p{p3’AusTemp} vs p{p3’AusSNP3G}, *p* = 0.0044; p{p3’AusTrop} vs p{p3’AusSNP3G}, *p* = 0.011]. (D-F) Shown are group averages for the splicing efficiency of dmpi8 (expressed as percentage of *dper* transcripts where the dmpi8 intron is spliced) throughout an LD cycle for three different genotypes of transgenic flies. Flies were entrained for five days of LD at the indicated temperatures, and collected on the fifth day by freezing at the indicated times. Extracts were prepared from isolated heads and dmpi8 splicing efficiency measured for each line separately, followed by pooling results from different lines to yield group averages for the tropical consensus, temperate consensus, and SNP3G version. For each temperature, the daily dmpi8 splicing curves were significantly different between the three different genotypes (one-sided Student’s *t-test*); [For 18°C; p{p3’AusTemp} vs p{p3’AusTrop}, *p* < 3.5 x 10^−4^; p{p3’AusTemp} vs p{p3’AusSNP3G}, *p* < 5.0 x 10^−7^; p{p3’AusTrop} vs p{p3’AusSNP3G}, *p* < 1.3 x 10^−11^], [For 25°C; p{p3’AusTemp} vs p{p3’AusTrop}, *p* = 0.013; p{p3’AusTemp} vs p{p3’AusSNP3G}, *p* < 1.9 x 10^−4^; p{p3’AusTrop} vs p{p3’AusSNP3G}, *p* < 2.4 x 10^−9^], [For 29°C; p{p3’AusTemp} vs p{p3’AusTrop}, *p* = 0.0061; p{p3’AusTemp} vs p{p3’AusSNP3G}, *p* < 1.5 x 10^−4^; p{p3’AusTrop} vs p{p3’AusSNP3G}, *p* < 9.9 x 10^−11^]. ZT, Zeitgeber time (hr).

At all temperatures tested, flies with the p3’AusTemp haplotype exhibited less daytime sleep levels compared to p{p3’AusTrop} flies (Fig 8A–8C). With regards to nighttime sleep, a similar trend of reduced total levels for p3’AusTemp was also observed, especially at higher temperatures, consistent with the temperate and tropical natural populations (Figs 1, 2 and 6). The p{p3’AusTemp} and p{p3’AusTrop} transgenic flies have similar ~24 hr free-running behavioral periods (S2 Table), consistent with the natural populations (S1 Table) and little effect of dmpi8 splicing on the pace of the clock [7, 8, 16]. Thus, there is strong correspondence between the results obtained using transgenic models and natural populations having the same temperate (T/T/C/A) and tropical (C/A/T/T) *dper* 3’ UTR sequence (Figs 6C and 8). Transgenic flies with the SNP3G variant exhibited less mid-day sleep then either the tropical or temperate major variants (Fig 8), further supporting the dominant effects of SNP3G irrespective of other *dper* 3’ UTR SNPs [12, 16] (Figs 6B and 7). At present, it is not clear why nighttime sleep levels in p{p3’AusSNP3G} are higher than those observed in p{p3’AusTemp} flies (Fig 8). With the SNP3G-containing transgenics we observed periods that are about 1 hr longer than the other genotypes, although the reason for this is presently not known (S2 Table).

The daily splicing efficiency of dmpi8 was significantly higher in transgenic flies with the T/T/C/A variant compared to C/A/T/T, and as expected both were lower than that observed with the SNP3G variant (Fig 8D–8F). Thus, there is strong correspondence between dmpi8 splicing efficiency and daytime sleep levels in the different transgenic models. These results identify novel naturally occurring *dper* 3’ UTR SNP combinations that affect sleep levels and dmpi8 splicing efficiency. Taken together, the findings obtained using transgenic models with the same genetic background provide direct functional evidence supporting the claim that the type of *dper* 3’ UTR haplotype makes significant contributions to why natural populations bearing the T/T/C/A variant exhibit less mid-day sleep compared to those with the C/A/T/T variant (Fig 6C). These findings are all internally consistent with the inverted directionality in the relative distributions of the T/T/C/A and C/A/T/T major haplotypes (Fig 5), and the overall tropical-temperate variation in the daytime sleep levels of natural populations of *D*. *melanogaster* along the eastern coast of Australia (Figs 1, 2, 6, S1 and S5).

## Discussion

Herein we analysed the daily wake-sleep profiles of 42 independent *D*. *melanogaster* populations from tropical and temperate regions representing the ‘tips’ of a well-studied latitudinal cline from the eastern coast of Australia [18, 22]. Monthly averages for the tropical regions range from 21–31 ^o^C and for the temperate region it is 8–16 ^o^C (www.worldweatheronline.com). The diverse climates that change along this latitudinal gradient have proven to be a good resource for studying local adaptation [20, 21]. These clines are thought to be associated with natural selection as a result of climatic variables, most notably ambient temperature [20, 21]. We show that over a wide range of temperatures, Australian flies from temperate regions have decreased daytime sleep levels compared to those from tropical regions (Figs 1–3, 6, S1, S4 and S5). This is strikingly similar to that observed in altitudinal clines from tropical Africa; i.e., flies adapted to high altitudes manifest less daytime sleep compared to their lowland counterparts [12]. The existence of parallel latitudinal and altitudinal clines further supports the notion that adjustments in the mid-day sleep levels of *Drosophila melanogaster* is a key aspect underlying the ability of this species to successfully colonize geographical regions with diverse temperature ranges.

Although flies from temperate regions of Australia and high altitudes in Africa exhibit decreased mid-day siesta, this behavior still shows thermal plasticity as daytime sleep levels increase with higher temperatures ([12] (Figs 1, 2, S1 and S4). While numerous factors likely contribute to the thermal changes in daytime sleep levels, an underlying factor is that the splicing efficiency of the dmpi8 intron is itself modulated by temperature [7]. The thermal sensitive nature of dmpi8 splicing is due to suboptimal 5’ and 3’ ss [8]. To date we have sequenced the *dper* 3’ UTR representing over 150 different natural populations from the Americas, Africa and Australia, and all have the identical suboptimal 5’ and 3’ss flanking the dmpi8 intron ([8, 12, 16]; manuscript in preparation, and see S1 Appendix). Thus, irrespective of the SNP content in the *dper* 3’ UTR, dmpi8 splicing remains relatively inefficient and temperature sensitive [16]. In addition, heat directly suppresses activity and/or induces sleep during the day [11, 29, 34, 35]. The combination of suboptimal splice sites on dmpi8 and the direct effects of heat ensure that the ability to mount a strong mid-day siesta on warm days is a conserved response in this species. As such, adaptation of *D*. *melanogaster* to the cooler climates beyond those typical of its ancestral range in the lowlands of equatorial Africa are not accompanied by a loss in the thermal plasticity of mid-day sleep. Rather, the relationship between mid-day siesta levels and ambient temperature appears to be differentially calibrated. Presumably, for *D*. *melanogaster* that adapted to regions with cooler daytime climates, selection for increased daytime activity when the risks from heat are reduced would seem advantageous. Thus, it is almost certain that the ability to mount a strong siesta in response to daytime heat reflects an ancestrally-based ‘fixed’ feature of all *D*. *melanogaster* strains, a response critical to survival. Conversely, the reduced mid-day siesta in temperate populations is a more recent adaptation to colonizing regions with cooler average daytime temperatures.

Prior studies of mid-day siesta as a function of altitude in African flies implicated a role for increased dmpi8 splicing efficiency in the successful colonization of high altitudes [12]. Likewise, temperate populations also show enhanced dmpi8 splicing efficiency compared to tropical populations along the eastern coast of Australia (Figs 4 and S3). These results make a compelling argument that dmpi8 splicing efficiency is a common mechanism targeted by natural selection in the clinal adaptation of mid-day siesta levels during the world-wide colonization of *D*. *melanogaster* beyond its ancestral range in the lowlands of Sub-Saharan Africa. In the case of African flies, we did not observe any spatial differences in *dper* 3’ UTR SNPS or haplotypes, indicating that other presently unknown factors underlie any altitudinal variations in dmpi8 splicing efficiency [12].

A novel aspect observed in Australia is that for a large portion of fly strains, but not all (e.g., SNP3G flies), thermal adaptation of mid-day sleep appears to include spatially varying selection of SNP variants in the *dper* 3’ UTR. Approximately half of the 42 independent populations analyzed in this study exhibit either the C/A/T/T or T/T/C/A haplotypes (Fig 5; S1 Appendix). Moreover, our interest was piqued by noting that these two major variants show an inverse relationship in their relative distributions between the temperate and tropical populations (Fig 5). Based on their respective distributions we predicted that the C/A/T/T haplotype should exhibit higher mid-day siesta levels compared to the T/T/C/A variant. Remarkably, this is what we observed in natural populations (Fig 6C), a result that was confirmed in transgenic models having the genetic background (Fig 8). Because the tropical-temperate variation in daytime sleep levels for Australian flies is similar to that observed in Africa for altitude [12], this multi-continental behavioral cline is almost certainly a *bona-fide* adaptation to temperature gradients. Therefore, the inverted geographical distributions of two major haplotypes in Australia that differentially calibrate mid-day siesta levels in a manner consistent with the cline, is also likely to reflect the outcome of natural selection. To the best of our knowledge, examples of reciprocal latitudinal variation in the frequency of common alleles that also have functional evidence linking them to an adaptive phenotype are rare. An example conceptually similar to our findings is the inverted latitudinal variation in the frequency of two common alleles along the Australian cline that control wing size in *Drosophila* [36].

Despite the tropical-temperate differences in the relative frequencies of the T/T/C/A and C/A/T/T haplotypes, a common limitation in these types of analyzes is that other mechanisms, most notably historical demographic events, can also lead to stable genetic differentiation among populations [37, 38]. For example, *D*. *melanogaster* in temperate regions of the United States and Australia are more closely related to European strains, whereas those in tropical regions are more related to African ancestry [37]. While we cannot rule out demography as a contributing factor for the inverse geographical distribution of the T/T/C/A and C/A/T/T haplotypes (see below), gene flow between *D*. *melanogaster* populations has been shown to be extensive and quite symmetrical along the Australian cline [39]. Indeed, the C/A/T/T and T/T/C/A haplotypes were predominant but not exclusive to tropical and temperate regions, respectively (Fig 5). Thus, even if demography played a major role in the initial establishment of clinal variation in genetic variants, the maintenance of an inverse temperate-tropical distribution for the T/T/C/A and C/A/T/T haplotypes suggests active selection.

Nonetheless, it would be highly informative to expand the number of populations analyzed and determine the distributions of the different *dper* 3’ UTR SNPs along the eastern coast of Australia at locations intermediate between the extreme ends of this latitudinal cline. In addition, sampling populations taken over many years could help determine if the tropical-temperate patterns in haplotype distribution and mid-day siesta are stable (e.g., [21]). Unfortunately, we were not able to obtain additional isofemale lines collected along the eastern coast of Australia as it appears those previously reported are no longer extant (C. Sgro, Monash University; personal communication). In addition, future studies will be required to determine the relative contributions of the *dper* 3’ UTR haplotype and other factors to daytime sleep levels in the natural populations of the C/A/T/T and T/T/C/A variants.

Our findings expand beyond SNP3 the repertoire of natural variations affecting dmpi8 splicing in the wild and highlights the possibility of co-evolving SNP combinations. Two of the SNPs (SNP2 and Af1) fall within the dmpi8 intron, whereas SNP1 and Af3 are positioned 5’ and 3’ to the intron, respectively (Fig 5). It is currently unclear why the C/A/T/T combination leads to reduced dmpi8 splicing efficiency compared to T/T/C/A (Figs 7 and 8). Possibilities include binding sites for splicing modulators, such as SR-proteins [40] and/or alternative RNA secondary structures [41]. In addition, it remains to be established what the relative dmpi8 splicing efficiencies are for the minor SNP3A variants observed in Australia (Fig 5).

All the SNPs observed in the *dper* 3’ UTR from the eastern coast of Australia are similar to those previously observed in cosmopolitan populations from the United States and more ancestral populations in Africa ([12, 16]; Fig 5, and manuscript in preparation). Likewise, all the natural variants of a Gly-Thr repeat found in the *dper* coding region of *D*. *melanogaster* from Australia were also present in more ancestral African populations [42]. These findings with *dper* are consistent with genome wide studies showing parallel geographic variation in flies from the United States and Australia that likely result from selection on standing ancestral variation [23]. Presumably, because *D*. *melanogaster* only arrived in the United States and Australia in the last 100–150 years [13, 14, 21], this might have limited the ability of any newly arising non-ancestral alleles to be prominent substrates for spatially varying selection [21]. Thus, a tentative conclusion at this point is that the different SNPs and SNP combinations in the *dper* 3’ UTRs currently observed in cosmopolitan strains reflect those originating in the ancestral African populations.

Intriguingly, the C/A/T/T and T/T/C/A variants are not as prominent along the eastern coast of the United States [16], whereas the VT1.1 and VT1.2 haplotypes common to the east coast of the United States were not observed along the eastern coast of Australia (Fig 5; S1 Appendix). It is possible that the early invasion and dispersal of *D*. *melanogaster* along the eastern coast of Australia might have originated with founding populations that contained a large abundance of flies bearing the C/A/T/T and T/T/C/A variants. Indeed, we speculate that the high abundance of the C/T/T/A and T/T/C/A haplotypes, along with the fact that these haplotypes yield significant differences in daytime sleep levels (Figs 6C and 8), might have ‘fortuitously’ made this pair attractive substrates for spatially varying selection along the Australian cline. We therefore view the inverted tropical-temperate distribution of the C/A/T/T and T/T/C/A variants as an additional ‘regional’ solution that works in conjunction with other, perhaps more globally shared, mechanisms contributing to clinal variation in dmpi8 splicing efficiency and/or mid-day siesta levels. The inverted distribution of C/A/T/T and T/T/C/A might add robustness or stability to the overall cline in mid-day siesta along the eastern coast of Australia.

Our findings are consistent with prior work showing that the 3’ UTR of *dper* has significant differentiation between the tropical and temperate populations of *D*. *melanogaster* in Australia (although the genomic changes were not identified) [22]. Why altitudinal variation in *dper* 3’ UTR SNPs is not observed in tropical Africa despite the presence of numerous haplotypes, including those found in Australia, is not clear. Whether other *dper* 3’ UTR haplotypes could also be good candidates for spatially varying selection is also not known. A more world-wide sampling of *dper* 3’ UTRs should be informative in addressing whether spatial variation in *dper* 3’ UTR SNPs is unique to the eastern coast of Australia, and might provide insights into the demographic histories surrounding the colonization of new regions by *D*. *melanogaster*. Clearly, a better understanding of this parallel thermal adaptation awaits the identity of factors besides *dper* 3’ UTR haplotypes that contribute to clinal variation in mid-day siesta levels and dmpi8 splicing efficiency. In this vein, we note that no latitudinal cline in daytime sleep levels or dmpi8 splicing efficiency was observed for *D*. *melanogaster* from the eastern coast of the United States, although nighttime sleep varied geographically [17]. This is not unique to mid-day sleep, as evidence based on *D*. *melanogaster* indicates that some traits show common clinal (altitude and/or latitude) variation between continents but not necessarily shared on all continents (e.g., [19]). It is possible that local calibration of mid-day siesta levels does not provide any fitness value for *D*. *melanogaster* along the eastern coast of the United States.

In summary, our findings indicate that the clinal adaptation of mid-day siesta has occurred several independent times on different continents. This includes altitudinal and latitudinal clines, strongly suggesting the main selection force is thermal gradients. It is likely that reduced mid-day siesta levels offers a selective advantage to *D*. *melanogaster* colonizing cooler temperatures where the risks from heat are diminished. The results herein strongly suggest that for a large portion of *D*. *melanogaster* from Australia, thermal adaptation along the eastern coast also involved spatial selection at the level of ancestrally derived SNPs in the *dper* 3’ UTR that differentially set dmpi8 splicing efficiency. While a parallel cline in mid-day sleep was also observed for flies from Africa as a function of elevation, and in some cases is linked to altitudinal differences in dmpi8 splicing efficiency, spatially varying SNPs in the *dper* 3’ UTR were not observed [12]. Therefore, even though a singular molecular mechanism appears to be a major evolutionary target (i.e., dmpi8 splicing efficiency) underlying a multi-continent parallel adaptation (i.e., mid-day siesta levels) that is driven by the same selective agent (i.e., temperature), there is regional variety in the solutions ‘found’ by natural selection despite shared genetic variation.

## Materials and methods

### Fly strains and general handling

All flies were routinely reared at room temperature (22–25°C) and maintained in vials or bottles containing standard agar-cornmeal-sugar-yeast-Tegosept-media. The natural populations of *D*. *melanogaster* based on independent isofemale lines that were used in this study were obtained from a collection maintained by Dr. D. Begun (UC Davis, USA), and are described in [18, 22] (see Fig 5 and S1 Appendix for a summary). The primary analysis was done using 8 lines from tropical and 8 lines from temperate populations, as follows: Tropical populations; (Cairns; 16.907 ^o^S, 145.709 ^o^E; HB22, HB25, HB27, HB106, HB108), (Cardwell; 18.267 ^o^S, 146.017 ^o^E; GT46, GT92, GT110); Temperate populations; (Sorell; 42.769 ^o^S, 147.576 ^o^E; S3, S4, S7, S8, S12, S22, S28, S34). We were then able to obtain an additional 26 lines, as follows; Tropical populations; (Cooktown; 15.476 ^o^S, 145.259 ^o^E; HF1, HF3, HF10, HF11, HF17, HF18, HF26, HF30, HF41), (Cairns; HB24, HB46), (Cardwell; GT18, GT21, GT24, GT77, GT91, GT112); Temperate populations (Miller’s Orchard; 41.237 ^o^S, 146.987 ^o^E; MIL2, MIL3, MIL4, MIL5, MIL6, MIL7, MIL8, MIL10), (Sorell; S17). The generation of transgenic flies is described below.

### Locomotor activity rhythms and sleep

Individual adult male or female flies (2–5 day-old) were placed in 65mm × 5mm glass tubes containing 5% sucrose with 2% Bacto agar. Locomotor activity was continuously monitored and recorded by using the Trikinetics (Waltham, MA, USA) system, as previously reported [27]. Briefly, throughout the testing period flies were maintained at the indicated temperature (18^o^, 25^o^ or 29°C) and subjected to at least 5 days of 12 hr light: 12 hr dark cycles [LD; where zeitgeber time 0 (ZT0) is defined as lights-on]. Cool white fluorescent light (~1000 lux) was used during LD and the temperature did not vary by more than 0.5°C between the light and dark periods. In general, after five days in LD, flies were kept in constant darkness (DD) or constant light (LL) for seven days. Data analysis of either locomotor activity or sleep parameters was done with the FaasX (kindly provide by Dr. F. Rouyer, Paris-Saclay Institute of Neuroscience, France) and MATLAB programs, as previously described [8, 27]. Sleep was defined as no detection of locomotor activity movement for any period of 5 contiguous min, which is routinely used in the field (e.g., [25]. When combining results from more than one independent *Drosophila* line to obtain group averages, the values were based on pooling data from the same number of individual flies (usually 16) for each line. Due to sexual dimorphism in daily sleep patterns, males and females were grouped separately. When calculating day/night values in 4-hour bins (e.g., Fig 2), if a sleep episode spans two neighboring bins, only its duration during a particular bin was used in the calculation for that particular bin.

In general, for measuring sleep values or locomotor activity, the data for LD was an average of the last three LD days. For DD or LL, the values were from single days. Free-running periods of locomotor activity rhythms was based on the data collected during six consecutive days in DD and using the FaasX program. P-values of significance were calculated by using Student’s *t*-test or ANOVA. Daily locomotor activity profiles were normalized such that the peak of evening activity was set to 1, facilitating visual comparison of the different transgenic genotypes, as previously described [8]. A correction was applied to neutralize “startle response” (i.e., increased bout of fly activity following the light-to-dark and dark-to-light environmental transitions) [6]; essentially the activity counts in the bin right after the environmental transition is replaced by an average of the activity counts in the bins just before and after.

### Sequencing of *dper* 3’ UTR

Genomic DNA was extracted from each of the 42 independent isofemale lines used in this study, followed by PCR with the primers P6754F (5’- TAGTCCACGATGCGATTCAATG-3’) and P7418R (5’-TGCAGCTAAATCCAATCCATC-3’). This yields a DNA fragment from position 6754 to nucleotide 7418 of the *dper* gene (numbering according to, [43]), which contains the *dper* 3’ UTR. The P6754F and P7418R primers were subsequently used for DNA sequencing of the PCR products in both directions, respectively. We did several independent sequencing studies. In one set, we used genomic DNA from a mixture of 10 male flies. In another set of studies, we used genomic DNA from individual male flies. For some lines, the *dper* 3’ UTR sequence was confirmed by sequencing from three separate males. Comparison of results from the population and individual flies yielded the identical *dper* 3’ UTR sequence for each isofemale line, and revealed that only one *dper* 3’ UTR sequence was present in each isofemale line. The sequences of the different *dper* 3’ UTRs for each natural population analysed in this manuscript is supplied as supplementary information (S1 Appendix).

### Generation of transgenic flies

To generate transgenic flies that produce *dper* transcripts with the desired 3’ UTR we used a previously described CaSpeR-4 based transformation vector containing a 13.2kb genomic *dper* insert that was modified by inserting sequences for KpnI and ApaI sites just upstream of the *dper* translation stop signal, termed CaSpeR13.2-KA [16]. The KpnI and ApaI sites are unique in the plasmid, facilitating the subcloning of different *per* 3’ UTRs. To generate the different *dper* 3’ UTR fragments for subcloning we used PCR in the presence of genomic DNA extracted from a natural population of Australian flies that carried the ‘temperate’ consensus, ‘tropical’ consensus, or a SNP3G variant. In addition, we introduced a 3xFLAG epitope tag just upstream of the *dper* translation stop codon. The PCR products were generated using the primers Kpn1_3FLAG_P6869 (5-’TATA***GGTACC***gagGCATGCGATTACAAGGATGACGACGATAAGGATTACAAGGATGACGACGATAAGGATTACAAGGATGACGACGATAAGtagtagccacacccgcag-3’) and P7371R (5’–GGGCGTTGGCTTTTCGATATTTATT- 3’). The Kpn1_3FLAG_P6889 primer has the following sequences from 5’ to 3’; KpnI site (bold italic), three spacer nucleotides (GAG), an Sph1 site (upper case, underlined), followed by the 3xflag sequence immediately upstream of the two TAG translation stop codons found on wildtype *dper*, followed by *dper* 3’ UTR sequences (lower case, underlined). This yields a DNA fragment which at the 5’ end has an in-frame Kpn1 site followed by an Sph1 site and 3xflag, natural *dper* translation stop codon, and sequences till position 7371 nt (numbering according to [43]). The PCR product was digested with Kpn1 and Bsu36I, followed by purification. The CaSpeR13.2-KA/VT1.1 backbone was also digested with Kpn1 and Bsu36I, purified and used to insert the desired *dper* 3’ UTR to yield the final transgenic constructs. The entire *dper* 3’UTR is ~500 bp long (depending on the haplotype) and the Bsu36I site is ~70 bp upstream of the cleavage/polyadenylation site. All the *dper* 3’ UTRs from Australian flies are identical downstream of the Bsu36 I site (see S1 Appendix). The entire *dper* 3’ UTR sequence was confirmed by DNA sequencing.

Transgenic flies were generated by Rainbow Transgenic Flies, Inc (Camarillo, CA, USA) using P-element transformation and injecting plasmids into a *w*^1118^ background. Recovered transgenic flies were subsequently crossed into a *w per*^0^ background with a double balancer line (*w per*^0^;*Sco*/*Cyo*;*MKRS*/*TM6B*), resulting in the transgenic lines termed; *wper*^0^; p{p3’AusTrop}, *w per*^0^; p{p3’AusTemp}, and *w per*^0^; p{p3’AusSNP3G}. Thus, the only functional version of *dper* in these transgenic flies is the transgene. At least three independent lines for each construct were obtained and analyzed, as follows: 1) p{p3’AusTrop}; HB22-f7, HB22-m21, HB22-m23, HB108-f31, HB108-f57R, HB108-f22, GT46-f4, GT46-f8, GT46-m5, GT92-f10Y, GT92-m9, GT92-m29; 2) p{p3’AusTemp}; S7-f25, S7-m16, S7-m21, S34-f11.2, S34-m9, S34-f5; 3) p{p3’AusSNP3G}, S4-f6, S4-f7, S4-m1.

### Splicing assay in flies

The splicing efficiency of dmpi8 in flies was measured as previously described [8, 32], with some minor modifications. Briefly, vials containing ~50 young (2- to 6-day-old) adult flies were placed in environmental chambers (Percival, USA) at the indicated temperature and exposed to at least five 12hr light: 12 hr dark cycles. At selected times during LD, flies were collected by freezing and heads isolated. Total RNA was extracted and the relative levels of *dper* mRNA either containing the dmpi8 intron or in which it was spliced measured using a semi-quantitative RT-PCR assay as previously described. In order to differentiate between the transgenic derived *dper* mRNA transcripts from the endogenously derived *per*^01^ transcripts we used the forward primers P6851m2F (5’-ACAGCACGGGGATGGG***GGTACC***-3’; KpnI site is in bold and italicized) and P6851 (5’ ACACAGCACGGGGATGGGTAGT 3’), respectively, as previously described [11, 12]. The P6851 primer will only amplify endogenously derived *dper* mRNA (e.g., *per*^01^ mRNA), whereas the P6851m2F primer will only amplify transgenic derived *dper* transcripts that contain the engineered Kpn1 site upstream of the *dper* translation stop codon. All RT-PCR reactions included the *dper* P7184r reverse primer (5’- GGCTTGAGATCTACATTATCCTC-3’); and the forward primer CBP294F (5’-TGATTGTGATGGGCCTGGACAAGT-3’) and reverse primer CBP536R (5’-GTCCAAGCGAGTGCCATTCACAAA-3’) to target the non-cycling Cap Binding Protein 20 (CBP20) gene as an internal control, as previously described [8, 32]. PCR products were separated and visualized by electrophoresis on 2% agarose gels containing Gelstar (Cambrex Co., USA), and the bands quantified using a Typhoon 9400 Imager. The values of *dper*-containing amplified products were normalized relative to CBP20 and expressed as either total *dper* mRNA or the proportion with the 3’-terminal intron removed. The relative abundance of total *dper* mRNA was calculated by adding the values for the two RT-PCR products; i.e., with and without the dmpi8 intron. We routinely collected samples after different PCR cycle lengths to ensure that the amplified products were in the linear range for quantification.

### Tissue culture constructs and dmpi8 splicing assay in S2 cell

A previously described construct, which contains the pAct5c promoter fused to a luciferase (luc) open reading frame fused to the entire *dper* 3’ UTR followed by approximately 90 bp of 3’ *dper* non-transcribed region (termed pAct-Luc-VT1.1) was used as the backbone vector to introduce the different 3’ UTRs from Australian flies [16]. To generate these constructs, genomic DNA from the natural populations was used as a template for PCR amplification in the presence of the primers StuI_P6869 (59-TA***AGGCC******T***AGTAGCCACACCCGCAGT-39) and P7371R (5’–GGGCGTTGGCTTTTCGATATTTATT- 3’) (The StuI site is in bold italic, immediately upstream of the *dper* translation stop codon; genomic *per* DNA sequences are underlined). The desired Australian *dper* 3’ UTRs were reconstructed into the pAct-Luc-VT1.1 vector by swapping the DNA fragment from the StuI site to the Bsu36I site in the *dper* 3’ UTR (see above, for transformation constructs) to yield the different vectors; pAct-Luc-p3’AusTrop, pAct-Luc-p3’AusTemp and pAct-Luc-p3’AusSNP3G. All final constructs used in this study were validated by DNA sequencing prior to their further use. Measurement of the dmpi8 splicing efficiency in *Drosophila* S2 cells was performed essentially as previously described [8, 16]. Briefly, the S2 cells and DES expression medium were purchased from Invitrogen, and S2 cells were transiently transfected using Effectene reagent (Qiagen), according to manufacturer’s instructions. Approximately 1.5 X 10^6^ S2 cells were placed in 6-well plates and transfected with 125 ng of the different vectors. After transfection, cells were allowed to recover for 2 days. Subsequently, cells were transferred to the indicated temperature for overnight incubation before harvesting. Cells were collected and washed twice with PBS on ice. Total RNA was extracted using the TRI Reagent LS (Sigma) according to manufacturer’s instructions. For each sample, about 1 *u*g of RNA was subjected to reverse transcription-PCR (RT-PCR) and dmpi8 splicing efficiency measured in the presence of the forward primer StuI_P6869 and the reverse primer P7184r, as previously described [8, 16].

## Supporting information

S1 Table Circadian values for daily activity rhythms in natural populations from Australia. (DOCX)

S2 Table Circadian values for daily activity rhythms in transgenic flies. (DOCX)

S1 Fig Tropical female flies exhibit increased daytime sleep compared to temperate populations (related to Figs 1 and 2). Adult female flies from 8 different tropical isofemale lines and 8 different temperate isofemale lines were kept at the indicated temperature (bottom of panels) and entrained for five days of 12 hr light/12 hr dark cycles (LD; where ZT0 is lights-on). For each isofemale line, the locomotor activity of individual flies (n = 32) was measured, followed by pooling the data to obtain a group average for the tropical and temperate populations. Shown is the total amount of sleep during either the 12 hr of day (A) or 12 hr of night (B), averaged over the last three days of LD. Values for tropical and temperate populations are significantly different using one-sided Student’s *t-test*; *, p < 0.05; **, p < 0.01. Fly lines used are as follows; tropical, HB22, HB25, HB27, HB106, HB108, GT46, GT92, GT110; temperate, S3, S4, S7, S8, S12, S22, S28, S34.
(TIF)

S2 Fig Tropical and temperate populations exhibit similar activity levels during wake periods (related to Figs 1 and 2). The results are based on the same flies and activity data used in Fig 1. Shown are group averages for activity levels (total number of beam crossings, i.e., counts) for each wake period, averaged over the last three days of LD. The following p values were determined (one-sided Student’s t-test); [18°C, day, *p* = 0.29; 18°C, night, *p* = 0.15; 25°C, day, *p* = 0.034; 25°C, night, *p* = 0.017; 29°C, day, *p* = 0.083; 29°C, night, *p* = 0.046]. Fly lines used are as follows; tropical, HB22, HB25, HB27, HB106, HB108, GT46, GT92, GT110; temperate, S3, S4, S7, S8, S12, S22, S28, S34.
(TIF)

S3 Fig Dmpi8 splicing efficiency is higher in temperate flies compared to tropical populations even during constant dark and constant light conditions (related to Fig 4). Adult flies from 8 different tropical isofemale lines and 8 different temperate isofemale lines were kept at 25°C and entrained for three days of 12 hr light/12 hr dark cycles (LD; where ZT0 is lights-on). Subsequently, the flies were split into two groups; one group was placed in constant darkness and collected during the first day (DD1; left panel), whereas the other group was placed in constant light and collected on the third day (LL3; right panel). Flies were collected at the indicated times (relative to the entraining LD cycle). Extracts were prepared from isolated heads and dmpi8 splicing efficiency measured for each line separately, followed by pooling results from different lines to yield group averages for the tropical and temperate populations. White, black, stripped, and light gray horizontal bars below panels represent 12-hr periods of light, dark, ‘subjective nighttime in LL, and ‘subjective daytime’ in DD, respectively. The daily dmpi8 splicing curves were significantly different between the tropical and temperate groups (one-way ANOVA); DD1, *p* = 0.0017; LL3, *p* = 0.00023. Fly lines used are as follows; tropical, HB22, HB25, HB27, HB106, HB108, GT46, GT92, GT110; temperate, S3, S4, S7, S8, S12, S22, S28, S34.
(TIF)

S4 Fig Analysis of an additional 26 independent Australian *D*. *melanogaster* populations further indicates that flies from temperate regions exhibit less daytime sleep compared to flies from tropical regions (related to Figs 1 and 6). **(**A-C) Adult male flies from an additional 17 independent tropical isofemale lines and 9 independent temperate isofemale lines were kept at the indicated temperature (top of panels) and entrained for five days of 12 hr light/12 hr dark cycles (LD; where ZT0 is lights-on). For each isofemale line, the locomotor activity of individual flies (n = 16 for each temperature) was measured, followed by pooling the data to obtain a group average for the tropical and temperate populations. The last three days’ worth of LD data was pooled, and shown are the daily sleep levels in 30 min bins. Fly lines used were as follows; tropical, GT18, GT21, GT24, GT77, GT91, GT112, HB24, HB46, HF1, HF3, HF10, HF11, HF17, HF18, HF26, HF30, HF41; temperate, MIL2, MIL3, MIL4, MIL5, MIL6, MIL7, MIL8, MIL10, S17. The gray shading in the panels represents dark periods. The results shown here are consistent with the original data using 8 tropical and 8 temperate populations (Fig 1). In Fig 6A is shown results obtained from the combined analysis of the 42 different natural populations analyzed in this study.
(TIF)

S5 Fig Total sleep time during day and night for each natural population of *D*. *melanogaster* analyzed in this study (related to Fig 6). Adult male flies were kept at 25°C and entrained for five days of 12 hr light/12 hr dark cycles (LD). For each isofemale line (42 in total), the locomotor activity of individual flies (n = 16) was measured, followed by pooling the data to obtain a group average for each population. Shown is the total amount of sleep (min) during either the 12 hr of day (top) or 12 hr of night (bottom), averaged over the last three days of LD. The data are the same as that used to generate Fig 6A.
(TIF)

S1 Appendix *dper* 3’ UTR sequences for the 42 independent Australian isofemale lines used in this study. The *dper* open reading frame has two sequential stop codons at the 3’ end. The DNA sequences below begin at the second stop signal and continue till the end of the *dper* 3’ UTR as annotated in flybase.org. According to the numbering cited in [43], the sequences shown below begin at position 6872 bp and end at 7368 bp. For each independent line, extracts were prepared from male flies, genomic DNA isolated, the *dper* 3’ UTR and flanking sequences amplified using PCR, followed by sequencing of the purified PCR product (see Materials and Methods). The dmpi8 sequence is highlighted in bold and underlined. A list of selected lines and their respective haplotypes are given at the end.
(DOCX)
