# Supplementary material for: Parallel clinal variation in the mid-day siesta of Drosophila melanogaster implicates continent-specific targets of natural selection
Source: PLoS Genet. 2018 Sep 4;14(9):e1007612. doi: 10.1371/journal.pgen.1007612 (PMC6138418; doi:10.1371/journal.pgen.1007612)
Supplement: S1 Table — (DOCX) [file pgen.1007612.s001.docx]

**Table S1. Circadian values for daily activity rhythms in natural populations from Australia**

| **Genotype^a^** | **Temperature** |  | **Period** | **Rhythmicty^c^** | **Power^d^** |
| --- | --- | --- | --- | --- | --- |
|  | **(°C)** | **n^b^** | **(hr ± sem)** | **(%)** | **(± sem)** |
| *HB22* | 18 | 12 | 23.4 ± 0.1 | 100 | 116.5 ± 18.8 |
|  | 25 | 30 | 24.3 ± 0.1 | 100 | 126 ± 10.9 |
|  | 29 | 13 | 24.8 ± 0.1 | 92.3 | 135.3 ± 14.5 |
| *HB25* | 18 | 13 | 23.6 ± 0.1 | 92.3 | 149.3 ± 14.9 |
|  | 25 | 30 | 24.4 ± 0.1 | 83.3 | 106.5 ± 7.7 |
|  | 29 | 12 | 24.2 ± 0.1 | 91.7 | 115.9 ± 11.2 |
| *HB27* | 18 | 15 | 22.6 ± 0.1 | 86.7 | 150.3 ± 19.8 |
|  | 25 | 28 | 23.9 ± 0.1 | 100 | 142.9 ± 10.6 |
|  | 29 | 12 | 24 ±0.1 | 100 | 118.1 ± 21.8 |
| *HB106* | 18 | 16 | 23.7 ± 0.1 | 100 | 176.2 ± 19.8 |
|  | 25 | 26 | 25 ± 0.1 | 96.2 | 121.8 ± 11.1 |
|  | 29 | 14 | 24.6 ±0.2 | 57.1 | 80.6 ± 12.4 |
| *HB108* | 18 | 16 | 23.2 ± 0.1 | 100 | 199.5 ± 15.7 |
|  | 25 | 30 | 24.2 ± 0.1 | 93.3 | 121.8 ± 8.3 |
|  | 29 | 13 | 23.6 ± 0.1 | 100 | 148.9 ± 14.8 |
| *GT46* | 18 | 10 | 23.7 ± 0.1 | 100 | 211.5 ± 28.9 |
|  | 25 | 27 | 23.9 ± 0.1 | 96.3 | 180.8 ± 13.1 |
|  | 29 | 14 | 24.2 ± 0.2 | 100 | 160.7 ± 19.7 |
| *GT92* | 18 | 15 | 23.6 ± 0.1 | 93.3 | 139.3 ± 15.8 |
|  | 25 | 27 | 24.4 ± 0.1 | 81.5 | 83.3 ± 6.2 |
|  | 29 | 16 | 24.6 ± 0.2 | 87.5 | 65.8 ± 10.2 |
| *GT110* | 18 | 6 | 23.6 ± 0.1 | 83.3 | 108.6 ± 28.0 |
|  | 25 | 27 | 24.7 ± 0.1 | 88.9 | 112.1 ± 9.8 |
|  | 29 | 10 | 25.2 ± 0.2 | 100 | 115.2 ± 16.4 |

| *Tropical populations (average)* | 18 | 103 | 23.4 ±0.1 | 95.1 | 160.9 ± 7.4 |
| --- | --- | --- | --- | --- | --- |
|  | 25 | 225 | 24.3 ± 0.1 | 92.4 | 125.6 ± 4.0 |
|  | 29 | 104 | 24.4 ±0.1 | 94 | 119.4 ± 6.3 |
| *S3* | 18 | 11 | 23.1 ± 0.2 | 81.8 | 97.3 ± 16.9 |
|  | 25 | 32 | 24 ± 0.1 | 90.6 | 138.3 ± 11.6 |
|  | 29 | 15 | 23.8 ± 0.1 | 86.7 | 137.1 ± 20.7 |
| *S4* | 18 | 9 | 23.6 ± 0.1 | 66.7 | 104.4 ± 20.1 |
|  | 25 | 30 | 23.9 ± 0.1 | 80 | 108.4 ± 11.0 |
|  | 29 | 15 | 23.6 ± 0.1 | 86.7 | 119.4 ± 14.0 |
| *S7* | 18 | 14 | 23.6 ± 0.2 | 85.7 | 97 ± 15.4 |
|  | 25 | 30 | 24.3 ± 0.1 | 73.3 | 92.4 ± 9.6 |
|  | 29 | 14 | 23.6 ± 0.1 | 85.7 | 127.5 ± 17.0 |
| *S8* | 18 | 14 | 23.5 ± 0.1 | 92.9 | 201.2 ± 16.6 |
|  | 25 | 30 | 24 ± 0.1 | 70 | 118.9 ± 13.6 |
|  | 29 | 11 | 23.6 ± 0.1 | 63.6 | 88.4 ± 18.4 |
| *S12* | 18 | 16 | 23.8 ± 0.2 | 81.3 | 100.5 ± 14.5 |
|  | 25 | 31 | 25.3 ± 0.2 | 80.6 | 97 ± 9.0 |
|  | 29 | 15 | 24.7 ± 0.1 | 86.7 | 86.1 ± 11.4 |
| *S22* | 18 | 16 | 23.8 ± 0.2 | 81.3 | 107.2 ± 10.7 |
|  | 25 | 31 | 23.9 ± 0.1 | 90.3 | 129.6 ± 9.0 |
|  | 29 | 15 | 23.4 ± 0.1 | 100 | 155.3 ± 14.0 |
| *S28* | 18 | 15 | 23.6 ± 0.1 | 80 | 112.1 ± 13.7 |
|  | 25 | 26 | 24.6 ± 0.2 | 73.1 | 79.5 ± 9.0 |
|  | 29 | 14 | 24.2 ± 0.1 | 92.9 | 169.8 ± 17.1 |
| *S34* | 18 | 16 | 23.5 ± 0.1 | 100 | 124.4 ± 16.0 |
|  | 25 | 32 | 24.1 ± 0.1 | 96.9 | 129.1 ± 8.7 |
|  | 29 | 16 | 23.6 ± 0.1 | 87.5 | 137.1 ± 19.3 |
| *Temperate populations*  *(average)* | 18 | 111 | 23.6 ± 0.1 | 84.7 | 120.4 ± 6.3 |
|  | 25 | 242 | 24.2 ± 0.1 | 82.2 | 114.1 ± 3.9 |
|  | 29 | 115 | 23.8 ± 0.1 | 87 | 130.6 ± 6.3 |
|  |  |  |  |  |  |

^a^Young male flies of the given genotype representing natural populations captured along the eastern coast of Australia were exposed to five days of 12:12LD at the indicated temperature, followed by 7 days in continuous darkness (DD). For each genotype, activity data collected during DD was averaged for each individual fly and then a group average was determined.

^b^n, number of flies used to calculate the values shown and that survived the entire testing period.

^c^Flies with a power value of greater than 10 and periods ≥ 20 and ≤ 30, were defined as rhythmic.

^d^Power, is a relative measure of the strength or amplitude of the rhythm in arbitrary units.
