# Supplementary material for: Parallel clinal variation in the mid-day siesta of Drosophila melanogaster implicates continent-specific targets of natural selection
Source: PLoS Genet. 2018 Sep 4;14(9):e1007612. doi: 10.1371/journal.pgen.1007612 (PMC6138418; doi:10.1371/journal.pgen.1007612)
Supplement: S2 Table — (DOCX) [file pgen.1007612.s002.docx]

**Table S2. Circadian values for daily activity rhythms in transgenic flies**

| **Genotype^a^** | **Temperature** |  | **Period** | **Rhythmicty^c^** | **Power^d^** |
| --- | --- | --- | --- | --- | --- |
|  | **(°C)** | **n^b^** | **(hr ± sem)** | **(%)** | **(± sem)** |
| *p{p3’AusTrop}* | 18 | 177 | 23.9 ± 0.1 | 92.7 | 180.8 ± 5.9 |
|  | 25 | 175 | 24.0 ± 0.1 | 97.7 | 259.5 ± 6.9 |
|  | 29 | 175 | 23.7 ± 0.1 | 98.9 | 295.3 ± 6.74 |
| *p{p3’AusTemp}* | 18 | 137 | 24.3 ± 0.1 | 92 | 197.6 ± 7.49 |
|  | 25 | 136 | 24.1 ± 0.1 | 97.1 | 272.5 ± 6.7 |
|  | 29 | 130 | 23.9 ± 0.1 | 100 | 330.6 ± 5.7 |
| *p{p3’AusSNP3G}* | 18 | 44 | 24.7 ± 0.1 | 93.2 | 153.1 ± 10.9 |
|  | 25 | 45 | 25.1 ± 0.1 | 100 | 278 ± 12.4 |
|  | 29 | 41 | 24.9 ±0.1 | 97.6 | 118.1 ± 21.8 |

^a^Young male flies of the given genotype were exposed to five days of 12:12LD at the indicated temperature, followed by 7 days in continuous darkness (DD). For each genotype, activity data during DD was averaged for each individual fly and then a group average was determined. For the transgenic flies, data from at least three independent lines was used.

^b^n, number of flies used to calculate the values shown and that survived the entire testing period.

^c^Flies with a power value of greater than 10 and period ≥ 20 and ≤ 30, were defined as rhythmic. ^d^Power, is a relative measure of the strength or amplitude of the rhythm in arbitrary units.
