# Supplementary figures and images for: Parallel clinal variation in the mid-day siesta of Drosophila melanogaster implicates continent-specific targets of natural selection
Source: PLoS Genet. 2018 Sep 4;14(9):e1007612. doi: 10.1371/journal.pgen.1007612 (PMC6138418; doi:10.1371/journal.pgen.1007612)

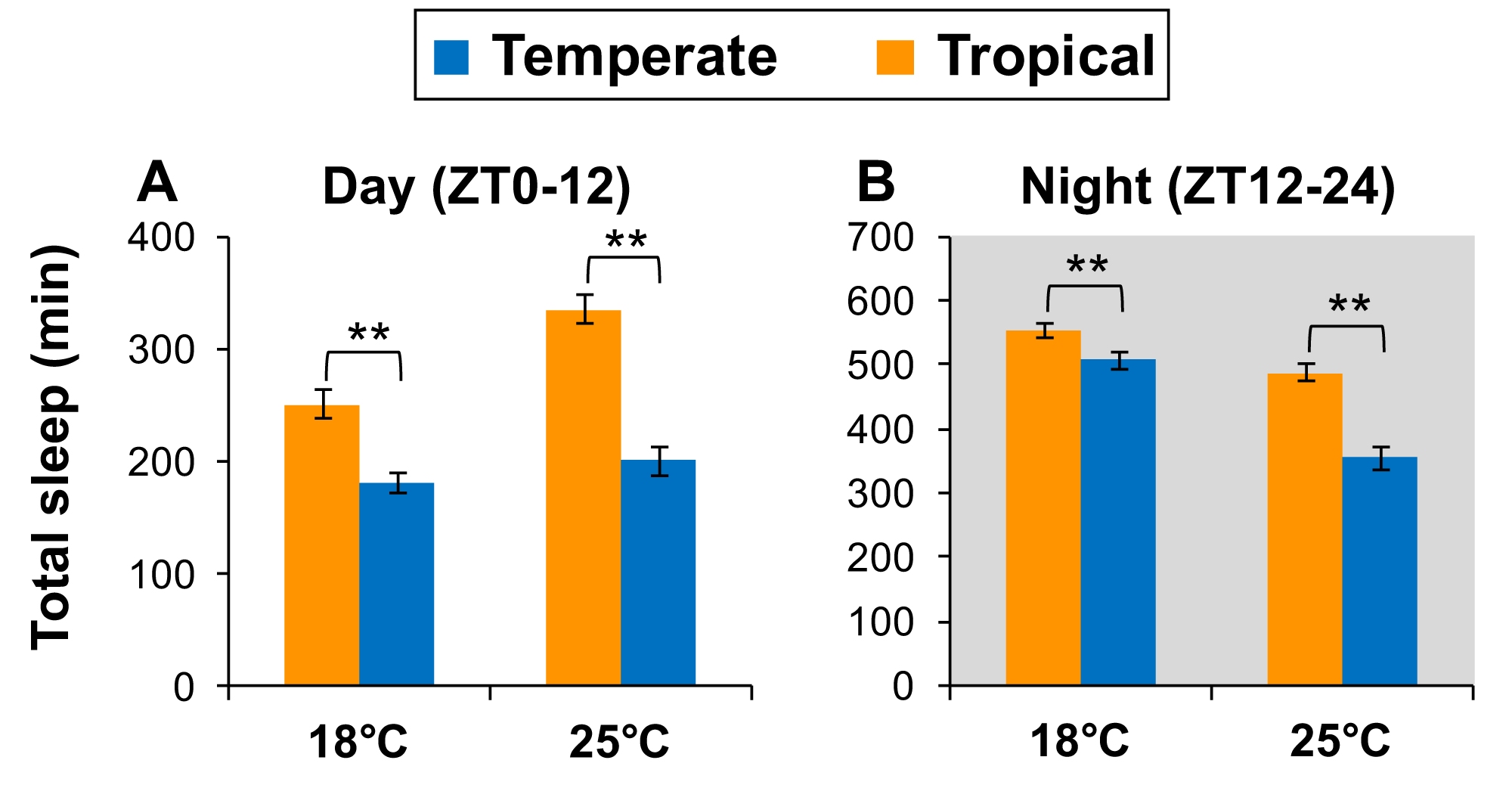

Supplement: S1 Fig — Adult female flies from 8 different tropical isofemale lines and 8 different temperate isofemale lines were kept at the indicated temperature (bottom of panels) and entrained for five days of 12 hr light/12 hr dark cycles (LD; where ZT0 is lights-on). For each isofemale line, the locomotor activity of individual flies (n = 32) was measured, followed by pooling the data to obtain a group average for the tropical and temperate populations. Shown is the total amount of sleep during either the 12 hr of day (A) or 12 hr of night (B), averaged over the last three days of LD. Values for tropical and temperate populations are significantly different using one-sided Student’s t-test; *, p < 0.05; **, p < 0.01. Fly lines used are as follows; tropical, HB22, HB25, HB27, HB106, HB108, GT46, GT92, GT110; temperate, S3, S4, S7, S8, S12, S22, S28, S34. (TIF) [file pgen.1007612.s003.tif]

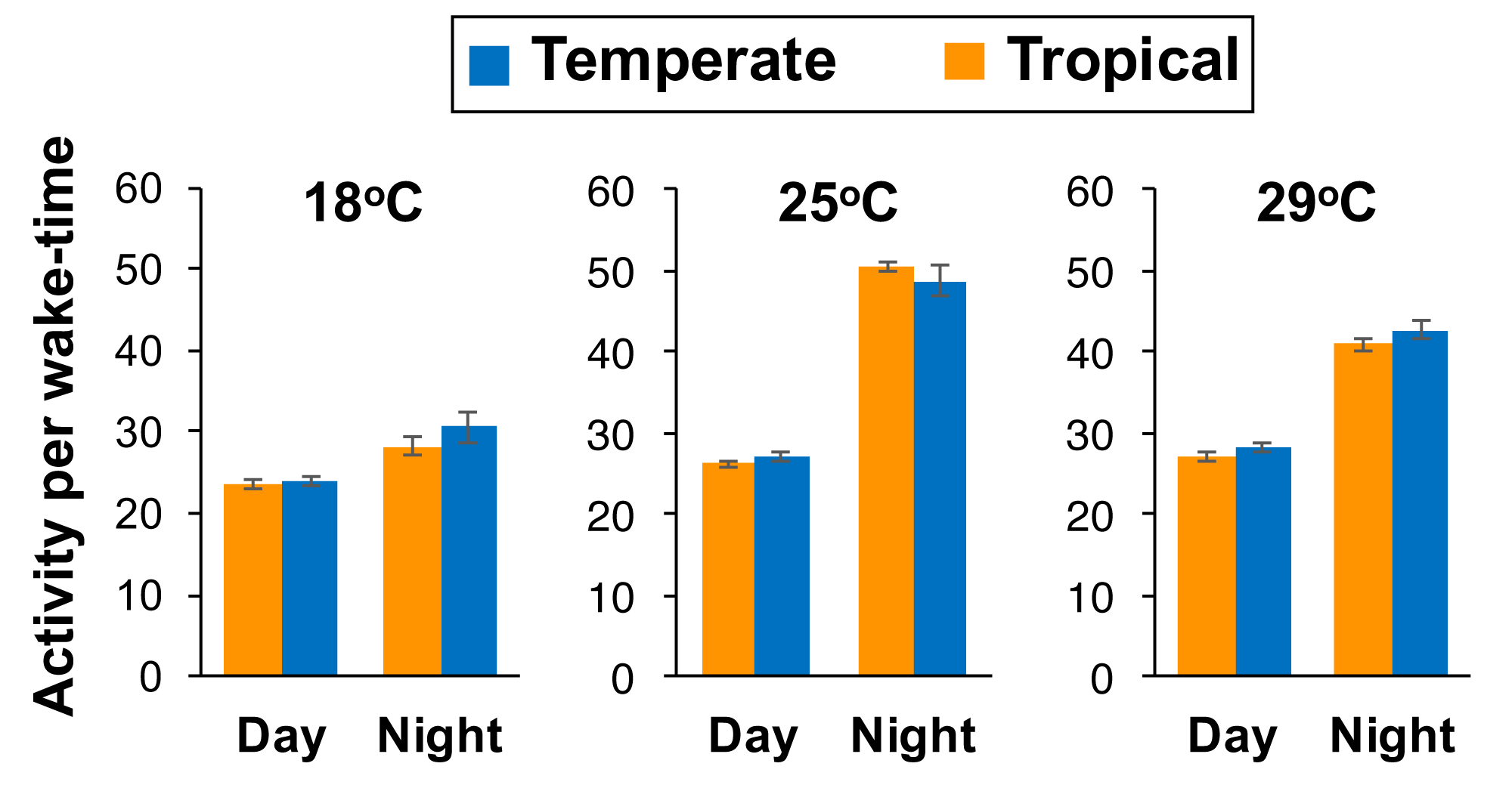

Supplement: S2 Fig — The results are based on the same flies and activity data used in Fig 1. Shown are group averages for activity levels (total number of beam crossings, i.e., counts) for each wake period, averaged over the last three days of LD. The following p values were determined (one-sided Student’s t-test); [18°C, day, p = 0.29; 18°C, night, p = 0.15; 25°C, day, p = 0.034; 25°C, night, p = 0.017; 29°C, day, p = 0.083; 29°C, night, p = 0.046]. Fly lines used are as follows; tropical, HB22, HB25, HB27, HB106, HB108, GT46, GT92, GT110; temperate, S3, S4, S7, S8, S12, S22, S28, S34. (TIF) [file pgen.1007612.s004.tif]

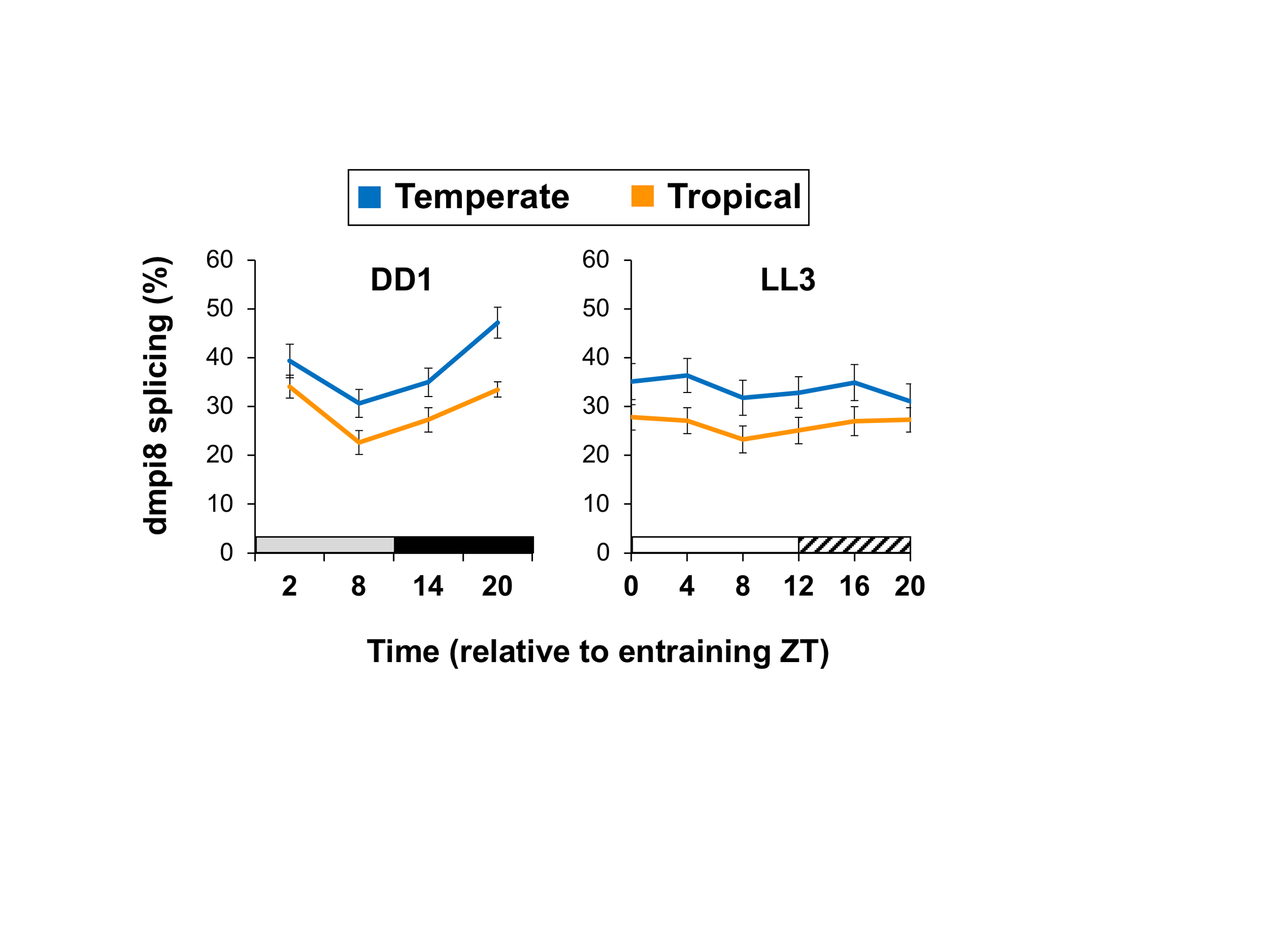

Supplement: S3 Fig — Adult flies from 8 different tropical isofemale lines and 8 different temperate isofemale lines were kept at 25°C and entrained for three days of 12 hr light/12 hr dark cycles (LD; where ZT0 is lights-on). Subsequently, the flies were split into two groups; one group was placed in constant darkness and collected during the first day (DD1; left panel), whereas the other group was placed in constant light and collected on the third day (LL3; right panel). Flies were collected at the indicated times (relative to the entraining LD cycle). Extracts were prepared from isolated heads and dmpi8 splicing efficiency measured for each line separately, followed by pooling results from different lines to yield group averages for the tropical and temperate populations. White, black, stripped, and light gray horizontal bars below panels represent 12-hr periods of light, dark, ‘subjective nighttime in LL, and ‘subjective daytime’ in DD, respectively. The daily dmpi8 splicing curves were significantly different between the tropical and temperate groups (one-way ANOVA); DD1, p = 0.0017; LL3, p = 0.00023. Fly lines used are as follows; tropical, HB22, HB25, HB27, HB106, HB108, GT46, GT92, GT110; temperate, S3, S4, S7, S8, S12, S22, S28, S34. (TIF) [file pgen.1007612.s005.tif]

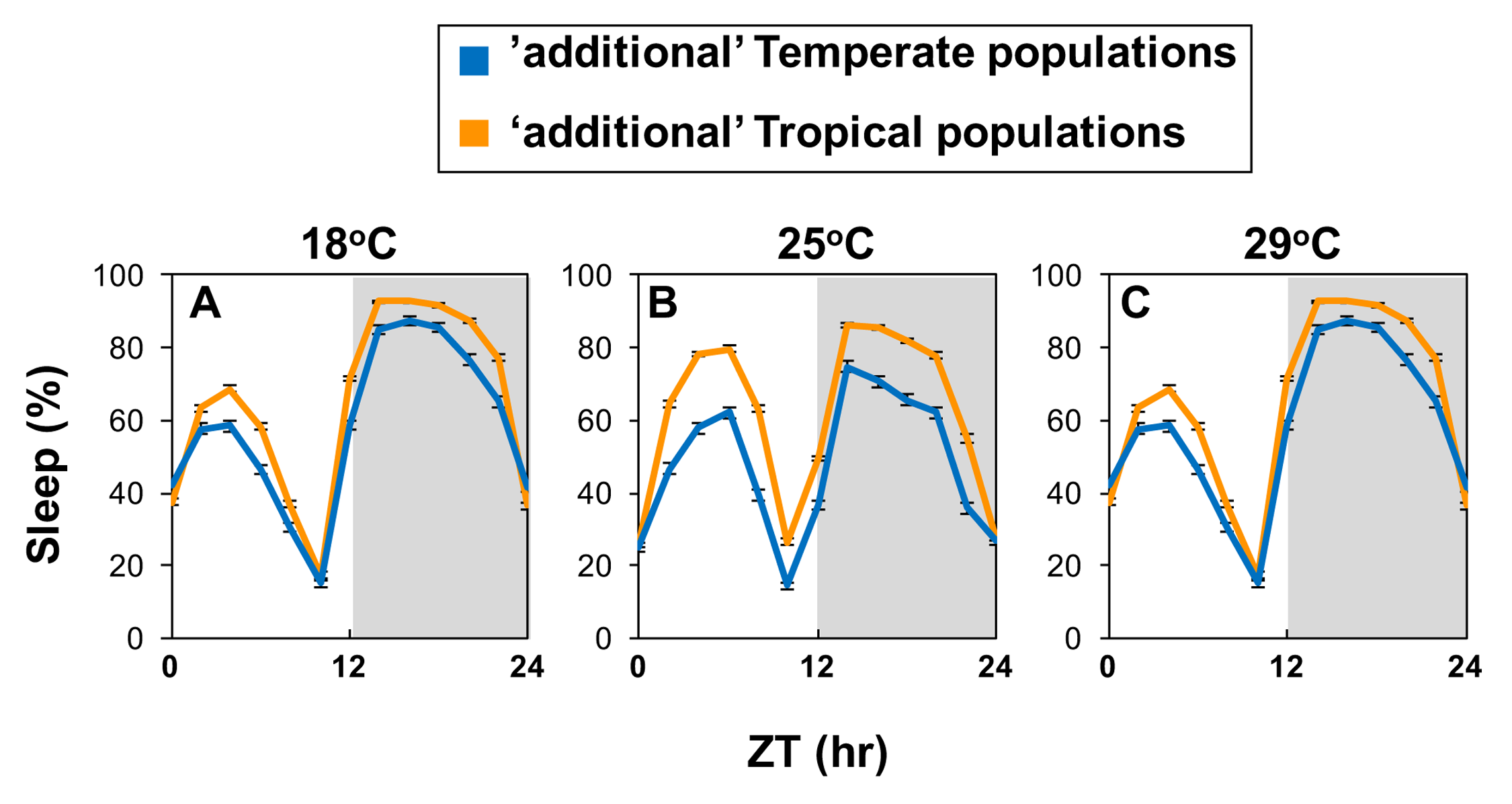

Supplement: S4 Fig — (A-C) Adult male flies from an additional 17 independent tropical isofemale lines and 9 independent temperate isofemale lines were kept at the indicated temperature (top of panels) and entrained for five days of 12 hr light/12 hr dark cycles (LD; where ZT0 is lights-on). For each isofemale line, the locomotor activity of individual flies (n = 16 for each temperature) was measured, followed by pooling the data to obtain a group average for the tropical and temperate populations. The last three days’ worth of LD data was pooled, and shown are the daily sleep levels in 30 min bins. Fly lines used were as follows; tropical, GT18, GT21, GT24, GT77, GT91, GT112, HB24, HB46, HF1, HF3, HF10, HF11, HF17, HF18, HF26, HF30, HF41; temperate, MIL2, MIL3, MIL4, MIL5, MIL6, MIL7, MIL8, MIL10, S17. The gray shading in the panels represents dark periods. The results shown here are consistent with the original data using 8 tropical and 8 temperate populations (Fig 1). In Fig 6A is shown results obtained from the combined analysis of the 42 different natural populations analyzed in this study. (TIF) [file pgen.1007612.s006.tif]

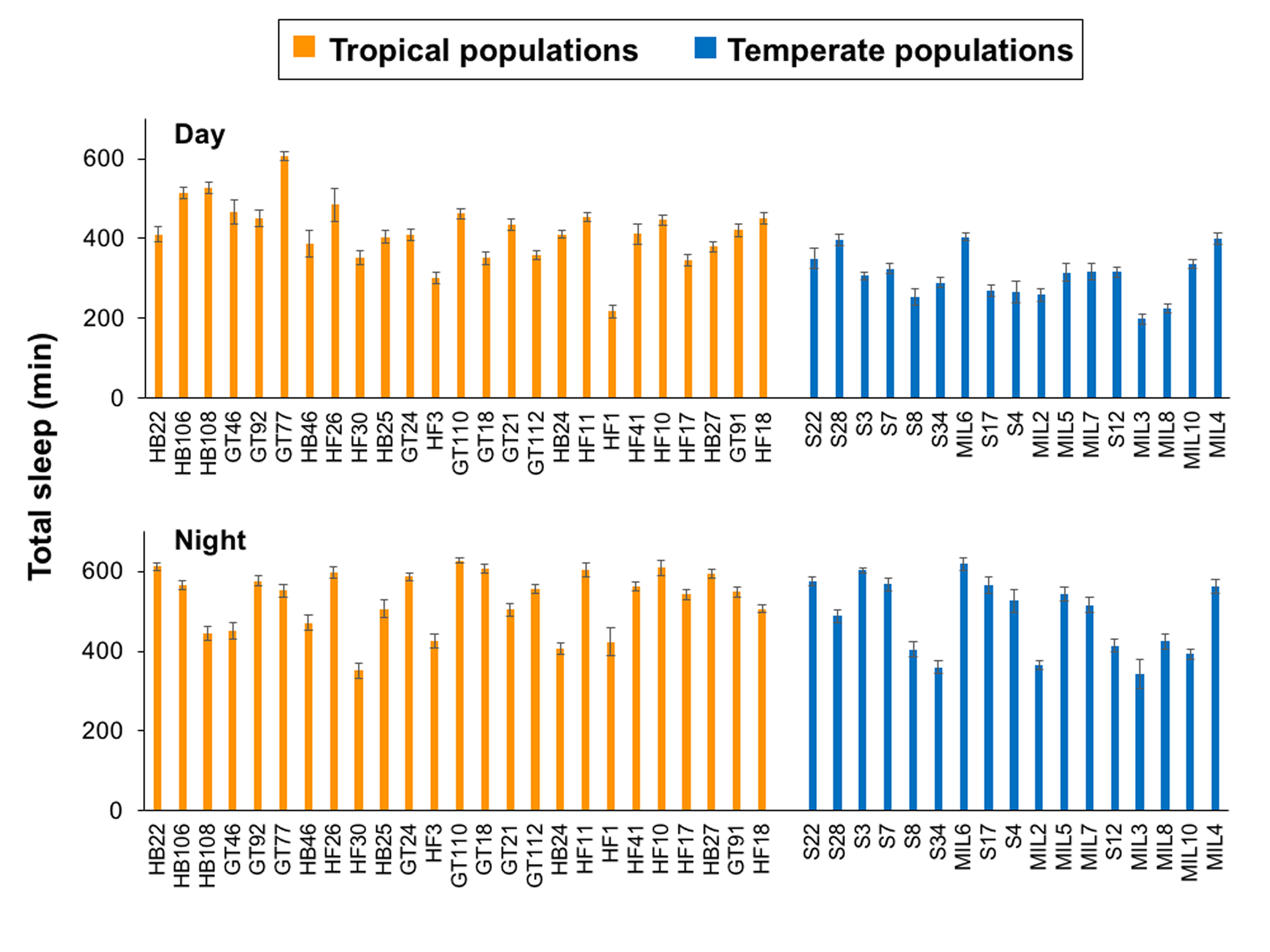

Supplement: S5 Fig — Adult male flies were kept at 25°C and entrained for five days of 12 hr light/12 hr dark cycles (LD). For each isofemale line (42 in total), the locomotor activity of individual flies (n = 16) was measured, followed by pooling the data to obtain a group average for each population. Shown is the total amount of sleep (min) during either the 12 hr of day (top) or 12 hr of night (bottom), averaged over the last three days of LD. The data are the same as that used to generate Fig 6A. (TIF) [file pgen.1007612.s007.tif]
