# Supplementary material for: Parallel clinal variation in the mid-day siesta of Drosophila melanogaster implicates continent-specific targets of natural selection
Source: PLoS Genet. 2018 Sep 4;14(9):e1007612. doi: 10.1371/journal.pgen.1007612 (PMC6138418; doi:10.1371/journal.pgen.1007612)
Supplement: S1 Appendix — The dper open reading frame has two sequential stop codons at the 3’ end. The DNA sequences below begin at the second stop signal and continue till the end of the dper 3’ UTR as annotated in flybase.org. According to the numbering cited in [43], the sequences shown below begin at position 6872 bp and end at 7368 bp. For each independent line, extracts were prepared from male flies, genomic DNA isolated, the dper 3’ UTR and flanking sequences amplified using PCR, followed by sequencing of the purified PCR product (see Materials and Methods). The dmpi8 sequence is highlighted in bold and underlined. A list of selected lines and their respective haplotypes are given at the end. (DOCX) [file pgen.1007612.s008.docx]

**Appendix S1: *dper* 3’ UTR sequences for Australian flies used in this study**

The *dper* open reading frame has two sequential stop codons at the 3’ end. The DNA sequences below begin at the second stop signal and continue till the end of the *dper* 3’ UTR as annotated in flybase.org. According to Citri et al. (Nature, 1987), the sequences shown below begin at position 6872 bp and end at 7368 bp. Sequencing was done as described in the Methods section. The dmpi8 sequence is highlighted in bold and underlined. A list of selected lines and their respective haplotypes are given at the end.

**Australia – Tropical populations**

**>HB22**

TAGCCACACCCGCAGTTGCTGCTGACCGACGTACACAACCGAGTGCACAATGTCGCTCCCTGGGACGATCCGAGCAGCAGACACCGAGGAGCAGCAGGGTCCTGGAAACGAGT**GAGCAATTGCCACCGGTCTGGGCACCGAGTACCAGCCAGTCCCACCAGTTCCCCCTATTCCTAACTCCCCCTTCCATTCCCTTCAAG**TTCTTCGAATCAACGCGAACGCTTCCGTGGGATGTCACCATTCAGTATCCGAACAAGTAACGAAAGCATCAGACAAACTGACGTGGGAGGATGCCCAGAACCCATGCAAGCCGAGGATAATGTAGATCTCAAGCCAAGCAAAGCTTTTAGATGATATCTATCCTATGTTTAATCAGAATATTAAGTGAATTGAAAACTAGAGTGGTTCGAACATCGAAGCACCCTTCTCAGCCCTAAGGTTTATATATCCGAATCTTTTTTAGTTATTTATTGTACAATAAATATCGAAAAGCCAAC

**>HB25**

TAGCCACACCCGCAGTTGCTGCTGACCGACGTACACAACCGAGTGCACAATGTCGATCCCTGGGATGATCCGAGCAGCAGACACCGAGGAGCAGCAGGGTCCTGGAAACGAGT**GAGCAATTGCCTCCGGTCTGGGCACCGAGCACCAGCCAGTCCCACCAGTTCCCCCTATTCCTAACTCCCCCTTCCATTCCCTTCAAG**TTCTTCGAATCAACGCGAACGCATCCGTGGGATGTCACCATTCAGTATCCGAACAAGTAACGAAAGCATCAGACAAACTGACGTGGGAGGATGCCCAGAACCCATGCAAGCCGAGGATAATGTAGATCTCAAGCCAAGCAAAGCTTTTAGATGATATCTATCCTATGTTTAATCAGAATATTAAGTGAATTGAAAACTAGAGTGGTTCGAACATCGAAGCACCCTTCTCAGCCCTAAGGTTTATATATCCGAATCTTTTTTAGTTATTTATTGTACAATAAATATCGAAAAGCCAAC

**>HB27**

TAGCCACACCCGCAGTTGCTGCTGACCGACGTACACAACCGAGTGCACAATGTCGATCCCTGGGACGATCCGAGCAGCAGACACCGAGGAGCAGCAGGGTCCTGGAAACGAGT**GAGCAATTGCCTCCGGTCTGGGCACCGAGCACCAGCCAGTCCCACCAGTTCCCCCTATTCCTAACTCCCCCTTCCATTCCCTTCAAG**TTCTTCGAATCAACGCGAACGCATCCGTGGGATGTCACCATTCAGTATCCGAACAAGTAACGAAAGCATCAGACAAACTGACGTGGGAGGATGCCCAGAACCCATGCAAGCCGAGGATAATGTAGATCTCAAGCCAAGCAAAGCTTTTAGATGATATCTATCCTATGTTTAATCAGAATATTAAGTGAATTGAAAACTAGAGTGGTTCGAACATCGAAGCACCCTTCTCAGCCCTAAGGTTTATATATCCGAATCTTTTTTAGTTATTTATTGTACAATAAATATCGAAAAGCCAAC

**>HB106**

TAGCCACACCCGCAGTTGCTGCTGACCGACGTACACAACCGAGTGCACAATGTCGATCCCTGGGACGATCCGAGCAGCAGACACCGAGGAGCAGCAGGGTCCTGGAAACGAGT**GAGCAATTGCCACCGGTCTGGGCACCGAGTACCAGCCAGTCCCACCAGTTCCCCCTATTCCTAACTCCCCCTTCCATTCCCTTCAAG**TTCTTCGAATCAACGCGAACGCTTCCGTGGGATGTCACCATTCAGTATCCGAACAAGTAACGAAAGCATCAGACAAACTGACGTGGGAGGATGCCCAGAACCCATGCAAGCCGAGGATAATGTAGATCTCAAGCCAAGCAAAGCTTTTAGATGATATCTATCCTATGTTTAATCAGAATATTAAGTGAATTGAAAACTAGAGTGGTTCGAACATCGAAGCACCCTTCTCAGCCCTAAGGTTTATATATCCGAATCTTTTTTAGTTATTTATTGTACAATAAATATCGAAAAGCCAAC

**>HB108**

TAGCCACACCCGCAGTTGCTGCTGACCGACGTACACAACCGAGTGCACAATGTCGATCCCTGGGACGATCCGAGCAGCAGACACCGAGGAGCAGCAGGGTCCTGGAAACGA**GTGAGCAATTGCCACCGGTCTGGGCACCGAGTACCAGCCAGTCCCACCAGTTCCCCCTATTCCTAACTCCCCCTTCCATTCCCTTCAAG**TTCTTCGAATCAACGCGAACGCTTCCGTGGGATGTCACCATTCAGTATCCGAACAAGTAACGAAAGCATCAGACAAACTGACGTGGGAGGATGCCCAGAACCCATGCAAGCCGAGGATAATGTAGATCTCAAGCCAAGCAAAGCTTTTAGATGATATCTATCCTATGTTTAATCAGAATATTAAGTGAATTGAAAACTAGAGTGGTTCGAACATCGAAGCACCCTTCTCAGCCCTAAGGTTTATATATCCGAATCTTTTTTAGTTATTTATTGTACAATAAATATCGAAAAGCCAAC

**>GT46**

TAGCCACACCCGCAGTTGCTGCTGACCGACGTACACAACCGAGTGCACAATGTCGATCCCTGGGACGATCCGAGCAGCAGACACCGAGGAGCAGCAGGGTCCTGGAAACGA**GTGAGCAATTGCCACCGGTCTGGGCACCGAGTACCAGCCAGTCCCACCAGTTCCCCCTATTCCTAACTCCCCCTTCCATTCCCTTCAAG**TTCTTCGAATCAACGCGAACGCTTCCGTGGGATGTCACCATTCAGTATCCGAACAAGTAACGAAAGCATCAGACAAACTGACGTGGGAGGATGCCCAGAACCCATGCAAGCCGAGGATAATGTAGATCTCAAGCCAAGCAAAGCTTTTAGATGATATCTATCCTATGTTTAATCAGAATATTAAGTGAATTGAAAACTAGAGTGGTTCGAACATCGAAGCACCCTTCTCAGCCCTAAGGTTTATATATCCGAATCTTTTTTAGTTATTTATTGTACAATAAATATCGAAAAGCCAAC

**>GT92**

TAGCCACACCCGCAGTTGCTGCTGACCGACGTACACAACCGAGTGCACAATGTCGATCCCTGGGACGATCCGAGCAGCAGACACCGAGGAGCAGCAGGGTCCTGGAAACGA**GTGAGCAATTGCCACCGGTCTGGGCACCGAGTACCAGCCAGTCCCACCAGTTCCCCCTATTCCTAACTCCCCCTTCCATTCCCTTCAAG**TTCTTCGAATCAACGCGAACGCTTCCGTGGGATGTCACCATTCAGTATCCGAACAAGTAACGAAAGCATCAGACAAACTGACGTGGGAGGATGCCCAGAACCCATGCAAGCCGAGGATAATGTAGATCTCAAGCCAAGCAAAGCTTTTAGATGATATCTATCCTATGTTTAATCAGAATATTAAGTGAATTGAAAACTAGAGTGGTTCGAACATCGAAGCACCCTTCTCAGCCCTAAGGTTTATATATCCGAATCTTTTTTAGTTATTTATTGTACAATAAATATCGAAAAGCCAAC

**>GT110**

TAGCCACACCCGCAGTTGCTGCTGACCGACGTACACAACCGAGTGCACAATGTCGATCCCTGGGATGATCCGAGCAGCAGACACCGAGGAGCAGCAGGGTCCTGGAAACGA**GTGAGCAATTGCCTCCGGTCTGGGCACCGAGTACCAGCCAGTCCCACCAGTTCCCCCTATTCCTAACTCCCCCTTCCATTCCCTTCAAG**TTCTTCGAATCAACGCGAACGCTTCCGTGGGATGTCACCATTCAGTATCCGAACAAGTAACGAAAGCATCAGACGAACTGACGTGGAAGGATGCCCAGAACCCATGCAAGCCGAGGATAATGTAGATCTCAAGCCAAGCAAAGCTTTTAGATGATATCTATCCTATGTTTAATCAGAATATTAAGTGAATTGAAAACTAGAGTGGTTCGAACATCGAAGCACCCTTCTCAGCCCTAAGGTTTATATATCCGAATCTTTTTTAGTTATTTATTGTACAATAAATATCGAAAAGCCAAC

**>GT18**

TAGCCACACCCGCAGTTGCTGCTGACCGACGTACACAACCGAGTGCACAATGTCGATCCCTGGGATGATCCGAGCAGCAGACACCGAGGAGCAGCAGGGTCCTGGAAAC**GAGTGAGCAATTGCCTCCGGTCTGGGCACCGAGTACCAGCCAGTCCCACCAGTTCCTATTCCTAACTCCCCCTTCCATTCCCTTCAAG**TTCTTCGAATCAACGCGAACGCTTCCGTGGGATGTCACCATTCAGTATCCGAACAAGTAACGAAAGCATCAGACGAACTGACGTGGAAGGATGCCCAGAACCCATGCAAGCCGAGGATAATGTAGATCTCAAGCCAAGCAAAGCTTTTAGATGATATCTATCCTATGTTTAATCAGAATATTAAGTGAATTGAAAACTAGAGTGGTTCGAACATCGAAGCACCCTTCTCAGCCCTAAGGTTTATATATCCGAATCTTTTTTAGTTATTTATTGTACAATAAATATCGAAAAGCCAAC

**>GT21**

TAGCCACACCCGCAGTTGCTGCTGACCGACGTACACAACCGAGTGCACAATGTCGATCCCTGGGATGATCCGAGCAGCAGACACCGAGGAGCAGCAGGGTCCTGGAAAC**GAGTGAGCAATTGCCTCCGGTCTGGGCACCGAGTACCAGCCAGTCCCACCAGTTCCTATTCCTAACTCCCCCTTCCATTCCCTTCAAG**TTCTTCGAATCAACGCGAACGCTTCCGTGGGATGTCACCATTCAGTATCCGAACAAGTAACGAAAGCATCAGACGAACTGACGTGGAAGGATGCCCAGAACCCATGCAAGCCGAGGATAATGTAGATCTCAAGCCAAGCAAAGCTTTTAGATGATATCTATCCTATGTTTAATCAGAATATTAAGTGAATTGAAAACTAGAGTGGTTCGAACATCGAAGCACCCTTCTCAGCCCTAAGGTTTATATATCCGAATCTTTTTTAGTTATTTATTGTACAATAAATATCGAAAAGCCAAC

**>GT24**

TAGCCACACCCGCAGTTGCTGCTGACCGACGTACACAACCGAGTGCACAATGTCGATCCCTGGGATGATCCGAGCAGCAGACACCGAGGAGCAGCAGGGTCCTGGAAAC**GAGTGAGCAATTGCCTCCGGTCTGGGCACCGAGCACCAGCCAGTCCCACCAGTTCCCCCTATTCCTAACTCCCCCTTCCATTCCCTTCAAG**TTCTTCGAATCAACGCGAACGCATCCGTGGGATGTCACCATTCAGTATCCGAACAAGTAACGAAAGCATCAGACAAACTGACGTGGGAGGATGCCCAGAACCCATGCAAGCCGAGGATAATGTAGATCTCAAGCCAAGCAAAGCTTTTAGATGATATCTATCCTATGTTTAATCAGAATATTAAGTGAATTGAAAACTAGAGTGGTTCGAACATCGAAGCACCCTTCTCAGCCCTAAGGTTTATATATCCGAATCTTTTTTAGTTATTTATTGTACAATAAATATCGAAAAGCCAAC

**>GT77**

TAGCCACACCCGCAGTTGCTGCTGACCGACGTACACAACCGAGTGCACAATGTCGATCCCTGGGACGATCCGAGCAGCAGACACCGAGGAGCAGCAGGGTCCTGGAAACGA**GTGAGCAATTGCCACCGGTCTGGGCACCGAGTACCAGCCAGTCCCACCAGTTCCCCCTATTCCTAACTCCCCCTTCCATTCCCTTCAAG**TTCTTCGAATCAACGCGAACGCTTCCGTGGGATGTCACCATTCAGTATCCGAACAAGTAACGAAAGCATCAGACAAACTGACGTGGGAGGATGCCCAGAACCCATGCAAGCCGAGGATAATGTAGATCTCAAGCCAAGCAAAGCTTTTAGATGATATCTATCCTATGTTTAATCAGAATATTAAGTGAATTGAAAACTAGAGTGGTTCGAACATCGAAGCACCCTTCTCAGCCCTAAGGTTTATATAGTCGAATCTTTTTTAGTTATTTATTGTACAATAAATATGAAAAGCCAAC

**>GT91**

TAGCCACACCCGCAGTTGCTGCTGACCGACGTACACAACCGAGTGCACAATGTCGATCCCTGGGACGATCCGAGCAGCAGACACCGAGGAGCAGCAGGGTCCTGGAAACGA**GTGAGCAATTGCCTCCGGTCTGGGCACCGAGCACCAGCCAGTCCCACCAGTTCCCCCTATTCCTAACTCCCCCTTCCATTCCCTTCAAG**TTCTTCGAATCAACGCGAACGCATCCGTGGGATGTCACCATTCAGTATCCGAACAAGTAACGAAAGCATCAGACAAACTGACGTGGGAGGATGCCCAGAACCCATGCAAGCCGAGGATAATGTAGATCTCAAGCCAAGCAAAGCTTTTAGATGATATCTAT CCTATGTTTAATCAGAATATTAAGTGAATTGAAAACTAGAGTGGTTCGAACATCGAAGCACCCTTCTCAGCCCTAAGGTTTATATATCCGAATCTTTTTTAGTTATTTATTGTACAATAAATATCGAAAAGCCAAC

**>GT112**

TAGCCACACCCGCAGTTGCTGCTGACCGACGTACACAACCGAGTGCACAATGTCGATCCCTGGGATGATCCGAGCAGCAGACACCGAGGAGCAGCAGGGTCCTGGAAACGA**GTGAGCAATTGCCTCCGGTCTGGGCACCGAGTACCAGCCAGTCCCACCAGTTCCCCCTATTCCTAACTCCCCCTTCCATTCCCTTCAAG**TTCTTCGAATCAACGCGAACGCTTCCGTGGGATGTCACCATTCAGTATCCGAACAAGTAACGAAAGCATCAGACGAACTGACGTGGAAGGATGCCCAGAACCCATGCAAGCCGAGGATAATGTAGATCTCAAGCCAAGCAAAGCTTTTAGATGATATCTATCCTATGTTTAATCAGAATATTAAGTGAATTGAAAACTAGAGTGGTTCGAACATCGAAGCACCCTTCTCAGCCCTAAGGTTTATATATCCGAATCTTTTTTAGTTATTTATTGTACAATAAATATCGAAAAGCCAAC

**>HB24**

TAGCCACACCCGCAGTTGCTGCTGACCGACGTACACAACCGAGTGCACAATGTCGATCCCTGGGATGATCCGAGCAGCAGACACCGAGGAGCAGCAGGGTCCTGGAAACGA**GTGAGCAATTGCCTCCGGTCTGGGCACCGAGTACCAGCCAGTCCCACCAGTTCCCCCTATTCCTAACTCCCCCTTCCATTCCCTTCAAG**TTCTTCGAATCAACGCGAACGCTTCCGTGGGATGTCACCATTCAGTATCCGAACAAGTAACGAAAGCATCAGACGAACTGACGTGGAAGGATGCCCAGAACCCATGCAAGCCGAGGATAATGTAGATCTCAAGCCAAGCAAAGCTTTTAGATGATATCTATCCTATGTTTAATCAGAATATTAAGTGAATTGAAAACTAGAGTGGTTCGAACATCGAAGCACCCTTCTCAGCCCTAAGGTTTATATATCCGAATCTTTTTTAGTTATTTATTGTACAATAAATATCGAAAAGCCAAC

**>HB46**

TAGCCACACCCGCAGTTGCTGCTGACCGACGTACACAACCGAGTGCACAATGTCGATCCCTGGGACGATCCGAGCAGCAGACACCGAGGAGCAGCAGGGTCCTGGAAACGA**GTGAGCAATTGCCACCGGTCTGGGCACCGAGTACCAGCCAGTCCCACCAGTTCCCCCTATTCCTAACTCCCCCTTCCATTCCCTTCAAG**TTCTTCGAATCAACGCGAACGCTTCCGTGGGATGTCACCATTCAGTATCCGAACAAGTAACGAAAGCATCAGACAAACTGACGTGGGAGGATGCCCAGAACCCATGCAAGCCGAGGATAATGTAGATCTCAAGCCAAGCAAAGCTTTTAGATGATATCTATCCTATGTTTAATCAGAATATTAAGTGAATTGAAAACTAGAGTGGTTCGAACATCGAAGCACCCTTCTCAGCCCTAAGGTTTATATATCCGAATCTTTTTTAGTTATTTATTGTACAATAAATATCGAAAAGCCAAC

**>HF1**

TAGCCACACCCGCAGTTGCTGCTGACCGACGTACACAACCGAGTGCACAATGTCGATCCCTGGGATGATCCGAGCAGCAGACACCGAGGAGCAGCAGGGTCCTGGAAACGAGT**GAGCAATTGCCTCCGGTCTGGGCACCGAGTACCAGCCAGTCCCACCAGTTCCCCCTATTCCTAACTCCCCCTTCCATTCCCTTCAAG**TTCTTCGAATCAACGCGAACGCTTCCGTGGGATGTCACCATTCAGTATCCGAACAAGTAACGAAAGCATCAGACGAACTGACGTGGAAGGATGCCCAGAACCCATGCAAGCCGAGGATAATGTAGATCTCAAGCCAAGCAAAGCTTTTAGATGATATCTATCCTATGTTTAATCAGAATATTAAGTGAATTGAAAACTAGAGTGGTTCGAACATCGAAGCACCCTTCTCAGCCCTAAGGTTTATATATCCGAATCTTTTTTAGTTATTTATTGTACAATAAATATCGAAAAGCCAAC

**>HF3**

TAGCCACACCCGCAGTTGCTGCTGACCGACGTACACAACCGAGTGCACAATGTCGATCCCTGGGATGATCCGAGCAGCAGACACCGAGGAGCAGCAGGGTCCTGGAAACGAGT**GAGCAATTGCCTCCGGTCTGGGCACCGAGCACCAGCCAGTCCCACCAGTTCCCCCTATTCCTAACTCCCCCTTCCATTCCCTTCAAG**TTCTTCGAATCAACGCGAACGCATCCGTGGGATGTCACCATTCAGTATCCGAACAAGTAACGAAAGCATCAGACAAACTGACGTGGGAGGATGCCCAGAACCCATGCAAGCCGAGGATAATGTAGATCTCAAGCCAAGCAAAGCTTTTAGATGATATCTATCCTATGTTTAATCAGAATATTAAGTGAATTGAAAACTAGAGTGGTTCGAACATCGAAGCACCATTCTCAGCCCTAAGGTTTATATATCCGAATCTTTTTTAGTTATTTATTGTACAATAAATATCGAAAAGCCAAC

**>HF10**

TAGCCACACCCGCAGTTGCTGCTGACCGACGTACACAACCGAGTGCACAATGTCGATCCCTGGGATGATCCGAGCAGCAGACACCGAGGAGCAGCAGGGTCCTGGAAACGAGT**GAGCAATTGCCACCGGTCTGGGCACCGAGTACCAGCCAGTCCCACCAGTTCCCCCTATTCCTAACTCCCCCTTCCATTCCCTTCAAG**TTCTTCGAATCAACGCGAACGCTTCCGTGGGATGTCACCATTCAGTATCCGAACAAGTAACGAAAGCATCAGACAAACTGACGTGGGAGGATGCCCAGAACCCATGCAAGCCGAGGATAATGTAGATCTCAAGCCAAGCAAAGCTTTTAGATGATATCTATCCTATGTTTAATCAGAATATTAAGTGAATTGAAAACTAGAGTGGTTCGAACATCGAAGCACCCTTCTCAGCCCTAAGGTTTATATATCCGAATCTTTTTTAGTTATTTATTGTACAATAAATATCGAAAAGCCAAC

**>HF11**

TAGCCACACCCGCAGTTGCTGCTGACCGACGTACACAACCGAGTGCACAATGTCGATCCCTGGGATGATCCGAGCAGCAGACACCGAGGAGCAGCAGGGTCCTGGAAACGAGT**GAGCAATTGCCTCCGGTCTGGGCACCGAGTACCAGCCAGTCCCACCAGTTCCTATTCCTAACTCCCCCTTCCATTCCCTTCAAG**TTCTTCGAATCAACGCGAACGCTTCCGTGGGATGTCACCATTCAGTATCCGAACAAGTAACGAAAGCATCAGACGAACTGACGTGGAAGGATGCCCAGAACCCATGCAAGCCGAGGATAATGTAGATCTCAAGCCAAGCAAAGCTTTTAGATGATATCTATCCTATGTTTAATCAGAATATTAAGTGAATTGAAAACTAGAGTGGTTCGAACATCGAAGCACCCTTCTCAGCCCTAAGGTTTATATATCCGAATCTTTTTTAGTTATTTATTGTACAATAAATATCGAAAAGCCAAC

**>HF17**

TAGCCACACCCGCAGTTGCTGCTGACCGACGTACACAACCGAGTGCACAATGTCGATCCCTGGGATGATCCGAGCAGCAGACACCGAGGAGCAGCAGGGTCCTGGAAACGAGT**GAGCAATTGCCACCGGTCTGGGCACCGAGTACCAGCCAGTCCCACCAGTTCCCCCTATTCCTAACTCCCCCTTCCATTCCCTTCAAG**TTCTTCGAATCAACGCGAACGCTTCCGTGGGATGTCACCATTCAGTATCCGAACAAGTAACGAAAGCATCAGACAAACTGACGTGGGAGGATGCCCAGAACCCATGCAAGCCGAGGATAATGTAGATCTCAAGCCAAGCAAAGCTTTTAGATGATATCTATCCTATGTTTAATCAGAATATTAAGTGAATTGAAAACTAGAGTGGTTCGAACATCGAAGCACCCTTCTCAGCCCTAAGGTTTATATATCCGAATCTTTTTTAGTTATTTATTGTACAATAAATATCGAAAAGCCAAC

**>HF18**

TAGCCACACCCGCAGTTGCTGCTGACCGACGTACACAACCGAGTGCACAATGTCGATCCCTGGGACGATCCGAGCAGCAGACACCGAGGAGCAGCAGGGTCCTGGAAACGAGT**GAGCAATTGCCTCCGGTCTGGGCACCGAGCACCAGCCAGTCCCACCAGTTCCCCCTATTCCTAACTCCCCCTTCCATTCCCTTCAAG**TTCTTCGAATCAACGCGAACGCATCCGTGGGATGTCACCATTCAGTATCCGAACAAGTAACGAAAGCATCAGACAAACTGACGTGGGAGGATGCCCAGAACCCATGCAAGCCGAGGATAATGTAGATCTCAAGCCAAGCAAAGCTTTTAGATGATATCTATCCTATGTTTAATCAGAATATTAAGTGAATTGAAAACTAGAGTGGTTCGAACATCGAAGCACCCTTCTCAGCCCTAAGGTTTATATATCCGAATCTTTTTTAGTTATTTATTGTACAATAAATATCGAAAAGCCAAC

**>HF26**

TAGCCACACCCGCAGTTGCTGCTGACCGACGTACACAACCGAGTGCACAATGTCGATCCCTGGGACGATCCGAGCAGCAGACACCGAGGAGCAGCAGGGTCCTGGAAACGAGT**GAGCAATTGCCACCGGTCTGGGCACCGAGTACCAGCCAGTCCCACCAGTTCCCCCTATTCCTAACTCCCCCTTCCATTCCCTTCAAG**TTCTTCGAATCAACGCGAACGCTTCCGTGGGATGTCACCATTCAGTATCCGAACAAGTAACGAAAGCATCAGACAAACTGACGTGGGAGGATGCCCAGAACCCATGCAAGCCGAGGATAATGTAGATCTCAAGCCAAGCAAAGCTTTTAGATGATATCTATCCTATGTTTAATCAGAATATTAAGTGAATTGAAAACTAGAGTGGTTCGAACATCGAAGCACCCTTCTCAGCCCTAAGGTTTATATATCCGAATCTTTTTTAGTTATTTATTGTACAATAAATATCGAAAAGCCAAC

**>HF30**

TAGCCACACCCGCAGTTGCTGCTGACCGACGTACACAACCGAGTGCACAATGTCGATCCCTGGGACGATCCGAGCAGCAGACACCGAGGAGCAGCAGGGTCCTGGAAACGAGT**GAGCAATTGCCACCGGTCTGGGCACCGAGTACCAGCCAGTCCCACCAGTTCCCCCTATTCCTAACTCCCCCTTCCATTCCCTTCAAG**TTCTTCGAATCAACGCGAACGCTTCCGTGGGATGTCACCATTCAGTATCCGAACAAGTAACGAAAGCATCAGACAAACTGACGTGGGAGGATGCCCAGAACCCATGCAAGCCGAGGATAATGTAGATCTCAAGCCAAGCAAAGCTTTTAGATGATATCTATCCTATGTTTAATCAGAATATTAAGTGAATTGAAAACTAGAGTGGTTCGAACATCGAAGCACCCTTCTCAGCCCTAAGGTTTATATATCCGAATCTTTTTTAGTTATTTATTGTACAATAAATATCGAAAAGCCAAC

**>HF41**

TAGCCACACCCGCAGTTGCTGCTGACCGACGTACACAACCGAGTGCACAATGTCGATCCCTGGAATGATCCGAGCAGCAGACACCGAGGAGCAGCAGGGTCCTGGAAACGAGT**GAGCAATTGCCTCCGGTCTGGGCACCGAGTACCAGCCAGTCCCACCAGTTCCCCCTATTCCTAACTCCCCCTTCCATTCCCTTCAAG**TTCTTCGAATCAACGCGAACGCTTCCGTGGGATGTCACCATTCAGTATCCGAACAAGTAACGAAAGCATCAGACGAACTGACGTGGAAGGATGCCCAGAACCCATGCAAGCCGAGGATAATGTAGATCTCAAGCCAAGCAAAGCTTTTAGATGATATCTATCCTATGTTTAATCAGAATATTAAGTGAATTGAAAACTAGAGTGGTTCGAACATCGAAGCACCCTTCTCAGCCCTAAGGTTTATATATCCGAATCTTTTTTAGTTATTTATTGTACAATAAATATCGAAAAGCCAAC

**Australia – Temperate populations**

**>S3**

TAGCCACACCCGCAGTTGCTGCTGACCGACGTACACAACCGAGTGCACAATGTCGATCCCTGGGATGATCCGAGCAGCAGACACCGAGGAGCAGCAGGGTCCTGGAAACGA**GTGAGCAATTGCCTCCGGTCTGGGCACCGAGCACCAGCCAGTCCCACCAGTTCCCCCTATTCCTAACTCCCCCTTCCATTCCCTTCAAG**TTCTTCGAATCAACGCGAACGCATCCGTGGGATGTCACCATTCAGTATCCGAACAAGTAACGAAAGCATCAGACAAACTGACGTGGGAGGATGCCCAGAACCCATGCAAGCCGAGGATAATGTAGATCTCAAGCCAAGCAAAGCTTTTAGATGATATCTATCCTATGTTTAATCAGAATATTAAGTGAATTGAAAACTAGAGTGGTTCGAACATCGAAGCACCCTTCTCAGCCCTAAGGTTTATATATCCGAATCTTTTTTAGTTATTTATTGTACAATAAATATCGAAAAGCCAAC

**>S4**

TAGCCACACCCGCAGTTGCTGCTGACCGACGTACACAACCGAGTGCACAATGTCGATCCCTGGGATGATCCGAGCAGCAGACACCGAGGAGCAGCAGGGTCCTGGAAACGA**GTGAGCAATTGCCTCCGGTCTGGGCACCGAGTACCAGCCAGTCCCACCAGTTCCTATTCCTAACTCCCCCTTCCATTCCCTTCAAG**TTCTTCGAATCAACGCGAACGCTTCCGTGGGATGTCACCATTCAGTATCCGAACAAGTAACGAAAGCATCAGACGAACTGACGTGGAAGGATGCCCAGAACCCATGCAAGCCGAGGATAATGTAGATCTCAAGCCAAGCAAAGCTTTTAGATGATATCTATCCTATGTTTAATCAGAATATTAAGTGAATTGAAAACTAGAGTGGTTCGAACATCGAAGCACCCTTCTCAGCCCTAAGGTTTATATATCCGAATCTTTTTTAGTTATTTATTGTACAATAAATATCGAAAAGCCAAC

**>S7**

TAGCCACACCCGCAGTTGCTGCTGACCGACGTACACAACCGAGTGCACAATGTCGATCCCTGGGATGATCCGAGCAGCAGACACCGAGGAGCAGCAGGGTCCTGGAAACGA**GTGAGCAATTGCCTCCGGTCTGGGCACCGAGCACCAGCCAGTCCCACCAGTTCCCCCTATTCCTAACTCCCCCTTCCATTCCCTTCAAG**TTCTTCGAATCAACGCGAACGCATCCGTGGGATGTCACCATTCAGTATCCGAACAAGTAACGAAAGCATCAGACAAACTGACGTGGGAGGATGCCCAGAACCCATGCAAGCCGAGGATAATGTAGATCTCAAGCCAAGCAAAGCTTTTAGATGATATCTATCCTATGTTTAATCAGAATATTAAGTGAATTGAAAACTAGAGTGGTTCGAACATCGAAGCACCCTTCTCAGCCCTAAGGTTTATATATCCGAATCTTTTTTAGTTATTTATTGTACAATAAATATCGAAAAGCCAAC

**>S8**

TAGCCACACCCGCAGTTGCTGCTGACCGACGTACACAACCGAGTGCACAATGTCGATCCCTGGGATGATCCGAGCAGCAGACACCGAGGAGCAGCAGGGTCCTGGAAACGA**GTGAGCAATTGCCTCCGGTCTGGGCACCGAGCACCAGCCAGTCCCACCAGTTCCCCCTATTCCTAACTCCCCCTTCCATTCCCTTCAAG**TTCTTCGAATCAACGCGAACGCATCCGTGGGATGTCACCATTCAGTATCCGAACAAGTAACGAAAGCATCAGACAAACTGACGTGGGAGGATGCCCAGAACCCATGCAAGCCGAGGATAATGTAGATCTCAAGCCAAGCAAAGCTTTTAGATGATATCTATCCTATGTTTAATCAGAATATTAAGTGAATTGAAAACTAGAGTGGTTCGAACATCGAAGCACCCTTCTCAGCCCTAAGGTTTATATATCCGAATCTTTTTTAGTTATTTATTGTACAATAAATATCGAAAAGCCAAC

**>S12**

TAGCCACACCCGCAGTTGCTGCTGACCGACGTACACAACCGAGTGCACAATGTCGATCCCTGGGACGATCCGAGCAGCAGACACCGAGGAGCAGCAGGGTCCTGGAAACGA**GTGAGCAATTGCCTCCGGTCTGGGCACCGAGCACCAGCCAGTCCCACCAGTTCCCCCTATTCCTAACTCCCCCTTCCATTCCCTTCAAG**TTCTTCGAATCAACGCGAACGCTTCCGTGGGATGTCACCATTCAGTATCCGAACAAGTAACGAAAGCATCAGACAAACTGACGTGGGAGGATGCCCAGAACCCATGCAAGCCGAGGATAATGTAGATCTCAAGCCAAGCAAAGCTTTTAGATGATATCTATCCTATGTTTAATCAGAATATTAAGTGAATTGAAAACTAGAGTGGTTCGAACATCGAAGCACCCTTCTCAGCCCTAAGGTTTATATATCCGAATCTTTTTTAGTTATTTATTGTACAATAAATATCGAAAAGCCAAC

**>S22**

TAGCCACACCCGCAGTTGCTGCTGACCGACGTACACAACCGAGTGCACAATGTCGATCCCTGGGACGATCCGAGCAGCAGACACCGAGGAGCAGCAGGGTCCTGGAAACGA**GTGAGCAATTGCCACCGGTCTGGGCACCGAGTACCAGCCAGTCCCACCAGTTCCCCCTATTCCTAACTCCCCCTTCCATTCCCTTCAAG**TTCTTCGAATCAACGCGAACGCTTCCGTGGGATGTCACCATTCAGTATCCGAACAAGTAACGAAAGCATCAGACAAACTGACGTGGGAGGATGCCCAGAACCCATGCAAGCCGAGGATAATGTAGATCTCAAGCCAAGCAAAGCTTTTAGATGATATCTATCCTATGTTTAATCAGAATATTAAGTGAATTGAAAACTAGAGTGGTTCGAACATCGAAGCACCCTTCTCAGCCCTAAGGTTTATATATCCGAATCTTTTTTAGTTATTTATTGTACAATAAATATCGAAAAGCCAAC

**>S28**

TAGCCACACCCGCAGTTGCTGCTGACCGACGTACACAACCGAGTGCACAATGTCGATCCCTGGGACGATCCGAGCAGCAGACACCGAGGAGCAGCAGGGTCCTGGAAACGA**GTGAGCAATTGCCACCGGTCTGGGCACCGAGTACCAGCCAGTCCCACCAGTTCCCCCTATTCCTAACTCCCCCTTCCATTCCCTTCAAG**TTCTTCGAATCAACGCGAACGCTTCCGTGGGATGTCACCATTCAGTATCCGAACAAGTAACGAAAGCATCAGACAAACTGACGTGGGAGGATGCCCAGAACCCATGCAAGCCGAGGATAATGTAGATCTCAAGCCAAGCAAAGCTTTTAGATGATATCTATCCTATGTTTAATCAGAATATTAAGTGAATTGAAAACTAGAGTGGTTCGAACATCGAAGCACCCTTCTCAGCCCTAAGGTTTATATATCCGAATCTTTTTTAGTTATTTATTGTACAATAAATATCGAAAAGCCAAC

**>S34**

TAGCCACACCCGCAGTTGCTGCTGACCGACGTACACAACCGAGTGCACAATGTCGATCCCTGGGATGATCCGAGCAGCAGACACCGAGGAGCAGCAGGGTCCTGGAAACGA**GTGAGCAATTGCCTCCGGTCTGGGCACCGAGCACCAGCCAGTCCCACCAGTTCCCCCTATTCCTAACTCCCCCTTCCATTCCCTTCAAG**TTCTTCGAATCAACGCGAACGCATCCGTGGGATGTCACCATTCAGTATCCGAACAAGTAACGAAAGCATCAGACAAACTGACGTGGGAGGATGCCCAGAACCCATGCAAGCCGAGGATAATGTAGATCTCAAGCCAAGCAAAGCTTTTAGATGATATCTATCCTATGTTTAATCAGAATATTAAGTGAATTGAAAACTAGAGTGGTTCGAACATCGAAGCACCCTTCTCAGCCCTAAGGTTTATATATCCGAATCTTTTTTAGTTATTTATTGTACAATAAATATCGAAAAGCCAAC

**>MIL2**

TAGCCACACCCGCAGTTGCTGCTGACCGACGTACACAACCGAGTGCACAATGTCGATCCCTGGGATGATCCGAGCAGCAGACACCGAGGAGCAGCAGGGTCCTGGAAACGAGT**GAGCAATTGCCTCCGGTCTGGGCACCGAGTACCAGCCAGTCCCACCAGTTCCTATTCCTAACTCCCCCTTCCATTCCCTTCAAG**TTCTTCGAATCAACGCGAACGCTTCCGTGGGATGTCACCATTCAGTATCCGAACAAGTAACGAAAGCATCAGACGAACTGACGTGGAAGGATGCCCAGAACCCATGCAAGCCGAGGATAATGTAGATCTCAAGCCAAGCAAAGCTTTTAGATGATATCTATCCTATGTTTAATCAGAATATTAAGTGAATTGAAAACTAGAGTGGTTCGAACATCGAAGCACCCTTCTCAGCCCTAAGGTTTATATATCCGAATCTTTTTTAGTTATTTATTGTACAATAAATATCGAAAAGCCAAC

**>MIL3**

TAGCCACACCCGCAGTTGCTGCTGACCGACGTACACAACCGAGTGCACAATGTCGATCCCTGGGACGATCCGAGCAGCAGACACCGAGGAGCAGCAGGGTCCTGGAAACGAGT**GAGCAATTGCCTCCGGTCTGGGCACCGAGCACCAGCCAGTCCCACCAGTTCCCCCTATTCCTAACTCCCCCTTCCATTCCCTTCAAG**TTCTTCGAATCAACGCGAACGCTTCCGTGGGATGTCACCATTCAGTATCCGAACAAGTAACGAAAGCATCAGACAAACTGACGTGGGAGGATGCCCAGAACCCATGCAAGCCGAGGATAATGTAGATCTCAAGCCAAGCAAAGCTTTTAGATGATATCTATCCTATGTTTAATCAGAATATTAAGTGAATTGAAAACTAGAGTGGTTCGAACATCGAAGCACCCTTCTCAGCCCTAAGGTTTATATATCCGAATCTTTTTTAGTTATTTATTGTACAATAAATATCGAAAAGCCAAC

**>MIL4**

TAGCCACACCCGCAGTTGCTGCTGACCGACGTACACAACCGAGTGCACAATGTCGATCCCTGGGACGATCCGAGCAGCAGACACCGAGGAGCAGCAGGGTCCTGGAAACGAGT**GAGCAATTGCCTCCGGTCTGGGCACCGAGCACCAGCCAGTCCCACCAGTTCCCCCTATTCCTAACTCCCCCTTCCATTCCCTTCAAG**TTCTTCGAATCAACGCGAACGCATCCGTGGGATGTCACCATTCAGTATCCGAACAAGTAACGAAAGCATCAGACAAACTGACGTGGGAGGATGCCCAGAACCCATGCAAGCCGAGGATAATGTAGATCTCAAGCCAAGCAAAGCTTTTAGATGATATCTATCCTATGTTTAATCAGAATATTAAGTGAATTGAAAACTAGAGTGGTTCGAACATCGAAGCACCCTTCTCAGCCCTAAGGTTTATATATCCGAATCTTTTTTAGTTATTTATTGTACAATAAATATCGAAAAGCCAAC

**>MIL5**

TAGCCACACCCGCAGTTGCTGCTGACCGACGTACACAACCGAGTGCACAATGTCGATCCCTGGGATGATCCGAGCAGCAGACACCGAGGAGCAGCAGGGTCCTGGAAACGAGT**GAGCAATTGCCTCCGGTCTGGGCACCGAGTACCAGCCAGTCCCACCAGTTCCTATTCCTAACTCCCCCTTCCATTCCCTTCAAG**TTCTTCGAATCAACGCGAACGCTTCCGTGGGATGTCACCATTCAGTATCCGAACAAGTAACGAAAGCATCAGACGAACTGACGTGGAAGGATGCCCAGAACCCATGCAAGCCGAGGATAATGTAGATCTCAAGCCAAGCAAAGCTTTTAGATGATATCTATCCTATGTTTAATCAGAATATTAAGTGAATTGAAAACTAGAGTGGTTCGAACATCGAAGCACCCTTCTCAGCCCTAAGGTTTATATATCCGAATCTTTTTTAGTTATTTATTGTACAATAAATATCGAAAAGCCAAC

**>MIL6**

TAGCCACACCCGCAGTTGCTGCTGACCGACGTACACAACCGAGTGCACAATGTCGATCCCTGGGATGATCCGAGCAGCAGACACCGAGGAGCAGCAGGGTCCTGGAAACGAGT**GAGCAATTGCCTCCGGTCTGGGCACCGAGCACCAGCCAGTCCCACCAGTTCCCCCTATTCCTAACTCCCCCTTCCATTCCCTTCAAG**TTCTTCGAATCAACGCGAACGCATCCGTGGGATGTCACCATTCAGTATCCGAACAAGTAACGAAAGCATCAGACAAACTGACGTGGGAGGATGCCCAGAACCCATGCAAGCCGAGGATAATGTAGATCTCAAGCCAAGCAAAGCTTTTAGATGATATCTATCCTATGTTTAATCAGAATATTAAGTGAATTGAAAACTAGAGTGGTTCGAACATCGAAGCACCCTTCTCAGCCCTAAGGTTTATATATCCGAATCTTTTTTAGTTATTTATTGTACAATAAATATCGAAAAGCCAAC

**>MIL7**

TAGCCACACCCGCAGTTGCTGCTGACCGACGTACACAACCGAGTGCACAATGTCGATCCCTGGGATGATCCGAGCAGCAGACACCGAGGAGCAGCAGGGTCCTGGAAACGAGT**GAGCAATTGCCTCCGGTCTGGGCACCGAGTACCAGCCAGTCCCACCAGTTCCTATTCCTAACTCCCCCTTCCATTCCCTTCAAG**TTCTTCGAATCAACGCGAACGCTTCCGTGGGATGTCACCATTCAGTATCCGAACAAGTAACGAAAGCATCAGACGAACTGACGTGGAAGGATGCCCAGAACCCATGCAAGCCGAGGATAATGTAGATCTCAAGCCAAGCAAAGCTTTTAGATGATATCTATCCTATGTTTAATCAGAATATTAAGTGAATTGAAAACTAGAGTGGTTCGAACATCGAAGCACCCTTCTCAGCCCTAAGGTTTATATATCCGAATCTTTTTTAGTTATTTATTGTACAATAAATATCGAAAAGCCAAC

**>MIL8**

TAGCCACACCCGCAGTTGCTGCTGACCGACGTACACAACCGAGTGCACAATGTCGATCCCTGGGACGATCCGAGCAGCAGACACCGAGGAGCAGCAGGGTNCTGGAAACGAGT**GAGCAATTGCCTCCGGTCTGGGCACCGAGCACCAGCCAGTCCCACCAGTTCCCCCTATTCCTAACTCCCCCTTCCATTCCCTTCAAG**TTCTTCGAATCAACGCGAACGCATCCGTGGGATGTCACCATTCAGTATCCGAACAAGTAACGAAAGCATCAGACAAACTGACGTGGGAGGATGCCCAGAACCCATGCAAGCCGAGGATAATGTAGATCTCAAGCCAAGCAAAGCTTTTAGATGATATCTATCCTATGTTTAATCAGAATATTAAGTGAATTGAAAACTAGAGTGGTTCGAACATCGAAGCACCCTTCTCAGCCCTAAGGTTTATATATCCGAATCTTTTTTAGTTATTTATTGTACAATAAATATCGAAAAGCCAAC

**>MIL10**

TAGCCACACCCGCAGTTGCTGCTGACCGACGTACACAACCGAGTGCACAATGTCGATCCCTGGGACGATCCGAGCAGCAGACACCGAGGAGCAGCAGGGTCCTGGAAACGAGT**GAGCAATTGCCTCCGGTCTGGGCACCGAGCACCAGCCAGTCCCACCAGTTCCCCCTATTCCTAACTCCCCCTTCCATTCCCTTCAAG**TTCTTCGAATCAACGCGAACGCATCCGTGGGATGTCACCATTCAGTATCCGAACAAGTAACGAAAGCATCAGACAAACTGACGTGGGAGGATGCCCAGAACCCATGCAAGCCGAGGATAATGTAGATCTCAAGCCAAGCAAAGCTTTTAGATGATATCTATCCTATGTTTAATCAGAATATTAAGTGAATTGAAAACTAGAGTGGTTCGAACATCGAAGCACCCTTCTCAGCCCTAAGGTTTATATATCCGAATCTTTTTTAGTTATTTATTGTACAATAAATATCGAAAAGCCAAC

**>S17**

TAGCCACACCCGCAGTTGCTGCTGACCGACGTACACAACCGAGTGCACAATGTCGATCCCTGGGATGATCCGAGCAGCAGACACCGAGGAGCAGCAGGGTCCTGGAAACGAGT**GAGCAATTGCCTCCGGTCTGGGCACCGAGCACCAGCCAGTCCCACCAGTTCCCCCTATTCCTAACTCCCCCTTCCATTCCCTTCAAG**TTCTTCGAATCAACGCGAACGCATCCGTGGGATGTCACCATTCAGTATCCGAACAAGTAACGAAAGCATCAGACAAACTGACGTGGGAGGATGCCCAGAACCCATGCAAGCCGAGGATAATGTAGATCTCAAGCCAAGCAAAGCTTTTAGATGATATCTATCCTATGTTTAATCAGAATATTAAGTGAATTGAAAACTAGAGTGGTTCGAACATCGAAGCACCCTTCTCAGCCCTAAGGTTTATATATCCGAATCTTTTTTAGTTATTTATTGTACAATAAATATCGAAAAGCCAAC

**List of fly lines sorted by *dper* 3’ UTR haplotype**

**SNP3A (30 lines): HB22, HB25, HB27, HB46, HB106, HB108, GT24, GT46, GT77, GT91, GT92, S3, S7, S8, S12, S17, S22, S28, S34, HF3, HF10, HF17, HF18, HF26, HF30, MIL3, MIL4, MIL6, MIL8, MIL10.**

**SNP3G (12 lines): HF1, HF11, HF41, HB24, GT18, GT21, GT110, GT112, S4, MIL2, MIL5, MIL7.**

**C/A/T/T (11 lines): HB22, HB46, HB106, HB108, GT46, GT77, GT92, HF26, HF30, S22, S28.**

**T/T/C/A (9 lines): HF3, GT24, HB25, S3, S7, S8, S17, S34, MIL6.**
